# Nov/CCN3 Enhances Cord Blood Engraftment by Rapidly Recruiting Latent Human Stem Cell Activity

**DOI:** 10.1016/j.stem.2020.02.012

**Published:** 2020-04-02

**Authors:** Rajeev Gupta, Virginia Turati, Duncan Brian, Craig Thrussel, Barry Wilbourn, Gillian May, Tariq Enver

**Affiliations:** 1 Stem Cell Group, UCL Cancer Institute, University College London, London WC1E 6BT, UK; 2 Manual Blood Sciences, Health Services Laboratories, The Halo Building, 1 Mabledon Place, London WC1H 9AX, UK; 3 Flow Cytometry Core Facility, UCL Cancer Institute, University College London, London WC1E 6BT, UK

**Keywords:** hematopoietic stem cell, umbilical cord blood transplantation, NOV (CCN3), HSC recruitment, ROS, C-MYC, hexokinase 2, stem cell state transition

## Abstract

Umbilical cord blood (UCB) has had considerable impact in pediatric stem cell transplantation, but its wider use is limited in part by unit size. Long-term *ex vivo* culture offers one approach to increase engraftment capacity by seeking to expand stem and progenitor cells. Here, we show brief incubation (8 h) of UCB CD34+ cells with the matricellular regulator Nov (CCN3) increases the frequency of serially transplantable hematopoietic stem cells (HSCs) 6-fold. This rapid response suggests recruitment rather than expansion of stem cells; accordingly, in single-cell assays, Nov increases the clonogenicity of phenotypic HSCs without increasing their number through cell division. Recruitment is associated with both metabolic and transcriptional changes, and tracing of cell divisions demonstrates that the increased clonogenic activity resides within the undivided fraction of cells. Harnessing latent stem cell potential through recruitment-based approaches will inform understanding of stem cell state transitions with implications for translation to the clinic.

## Highlights

• NOV rapidly increases the number of functional HSCs in a single cord blood unit
• This is by direct recruitment without expansion or self-renewal *ex vivo*
• NOV reduces C-MYC and ROS but increases glycolytic enzymes in HSCs
• Manipulating non-dividing stem cells can alter their state and functional potential

## Graphical Abstract

**Figure:**
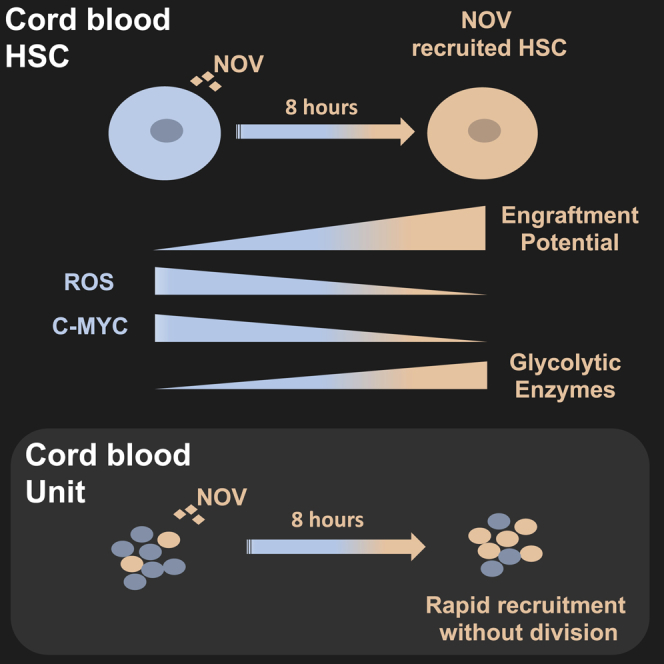

## Teaser

Umbilical cord blood (UCB) HSCs offer several clinical advantages, but many units are too small for use. NOV rapidly increases the number of functional HSCs in single UCB units by recruitment rather than expansion. Manipulation of stem cell state transitions offers an alternative approach to enhance HSC activity.

## Introduction

Allogeneic hematopoietic stem cell (HSC) transplantation is an important therapy for many blood disorders, and for certain diseases, it is the treatment of choice. As a source of HSCs, umbilical cord blood (UCB) has the advantage of requiring less stringent cross-matching together with a lower incidence of graft versus host disease (Broxmeyer, 2016).

UCB is widely used in pediatric stem cell transplantation, but units contain relatively few HSCs, limiting their use in older children and adults. Cell dose is a key determinant of clinical outcome (Brunstein and Wagner, 2006), and sub-optimal doses increase the risk of both infectious complications and graft failure. Administering 2 UCB units can address this but presents additional challenges in respect of costs, logistics, and understanding of the dynamics of durable long-term reconstitution, and it does not reduce transplant-related mortality (Bhella et al., 2018). Attention has therefore focused on strategies to maximize the benefit of a single UCB unit by increasing the numbers of functional HSCs. UCB-derived HSCs are the best characterized human stem cells; their homing and engraftment characteristics have been studied extensively in xenograft models (e.g., Doulatov et al., 2012 and references therein). This underlying biological understanding provides the framework for attempts to improve UCB transplantation by manipulating HSCs *ex vivo* to enhance their function. Conceptually, UCB transplantation could be improved by (1) collecting and processing UCB under conditions that better preserve HSC function *ex vivo* (e.g., hypoxia; Mantel et al., 2015), (2) enhancing the survival of HSCs or their homing to recipient bone marrow (reviewed by Broxmeyer, 2016 and Nikiforow and Ritz, 2016), (3) increasing total numbers of HSCs by enforcing self-renewal divisions *ex vivo*, or (4) increasing the potential of HSCs to engraft.

Prospectively isolated HSCs appear dormant or quiescent *ex vivo*, and compartments with the highest LT (long-term) HSC function contain cells that are slow to divide in culture, taking up to 80 h to first division (Morrison and Weissman, 1994, Cheung and Rando, 2013, Laurenti et al., 2015). They are characterized by low levels of both c-MYC and reactive oxygen species (ROS) (Cabezas-Wallscheid et al., 2017 and references therein), enhanced glycolysis (Ito and Suda, 2014), coordinated stress response programs (Xie et al., 2019), and lower rates of transcription (Freter et al., 2010) and translation (Signer et al., 2014). Although perhaps counterintuitive, given these biological properties, several strategies to increase UCB HSC numbers by forced self-renewal *ex vivo* prior to transplantation have been explored. They typically involve several days’ incubation with cytokines (most often “STF”; stem cell factor (SCF), thrombopoietin (TPO), Flt3 ligand), together with small molecules or other cytokines, which may either suppress differentiation or increase self-renewal in dividing HSCs (e.g., Boitano et al., 2010, Delaney et al., 2010, Fares et al., 2014, Guo et al., 2018).

Comparing the efficacy of different strategies is complicated by the complexity and retrospective nature of the methodologies used to enumerate HSCs in xenograft models. Direct comparison of the numbers and frequencies of engrafting cells in the starting material and the expanded product can also be difficult. Nevertheless, these techniques have increased short-term (ST) HSCs as scored in primary recipients, although their impact on the numbers of LT-HSCs scored in secondary recipients is sometimes less clear. Moreover, the key question of to what extent the agents used in expansion protocols improve performance over unmanipulated cells from the same UCB unit, or merely arrest a decay in HSC function that occurs due to prolonged *ex vivo* culture, can be challenging to evaluate.

Consistent with studies in xenograft models, in early-phase clinical trials, expanded UCB products generally alleviate the clinical problem of delayed early reconstitution but have less impact on long-term reconstitution (Wagner et al., 2016). *Ex vivo* expansion is both expensive and challenging; an alternative approach is to increase the functionality—rather than number—of HSCs in a UCB unit. In most transplant settings, it is likely that not all HSCs present can or will engraft. Indeed, the frequency of functional HSCs is at best 50% within the phenotypically defined UCB compartments that are most highly enriched in HSC activity (Majeti et al., 2007, Notta et al., 2011). Although in part due to limitations of both xenograft assays and HSC enrichment strategies (Knapp et al., 2018), this may also reflect the heterogeneity of HSCs and the probabilistic nature of their fate decisions (Roeder and Lorenz, 2006) and suggests untapped transplantation potential in UCB units.

We have previously demonstrated that (1) the matricellular regulator NOV is essential for primary engraftment of UCB-derived CD34+ cells, (2) its enforced expression enhances secondary engraftment, and (3) soluble NOV rescues some functional defects in human HSCs in which NOV has been knocked down (Gupta et al., 2007). Furthermore, NOV synergizes with TPO to maintain mouse HSCs (Ishihara et al., 2014), signals through several key pathways active in HSCs (reviewed in Li et al., 2015), displays anti-proliferative properties in other cell types (Bleau et al., 2007), and preserves stem cell clonogenicity better than STF alone in 10-day cultures of human progenitors (Gupta et al., 2007). Based on these observations, we explored whether soluble NOV might find utility in strategies to increase the long-term engraftment potential of UCB.

Here, we show that soluble NOV marks phenotypic LT-HSCs and increases the frequency of serially transplantable HSCs 6-fold. Furthermore, when a single freshly thawed UCB unit is tested by transplantation both before and directly after exposure to NOV, engraftment is increased. Strikingly, these effects require only an 8-h exposure and are independent of cell division *ex vivo*.

At the molecular level, NOV treatment leads to diminished expression of c-Myc protein and lower ROS levels, as well as increased transcription of genes encoding glycolytic enzymes. These effects suggest rapid functional recruitment of HSCs, which we formally demonstrate *in vitro* using a single-cell approach.

Our studies support the principle that recruitment of otherwise non-functioning HSCs can enhance UCB transplantation without the requirement for prolonged cell culture, underscoring the therapeutic potential of this approach.

## Results

### NOV Marks Phenotypic HSCs in UCB

The UCB CD34+ compartment may be divided by surface immunophenotyping into sub-populations, each with characteristic frequencies of cells able to score in the various *in vitro* clonogenic and *in vivo* transplantation assays that define stem and progenitor function experimentally (Doulatov et al., 2012).

**Figure 1:**
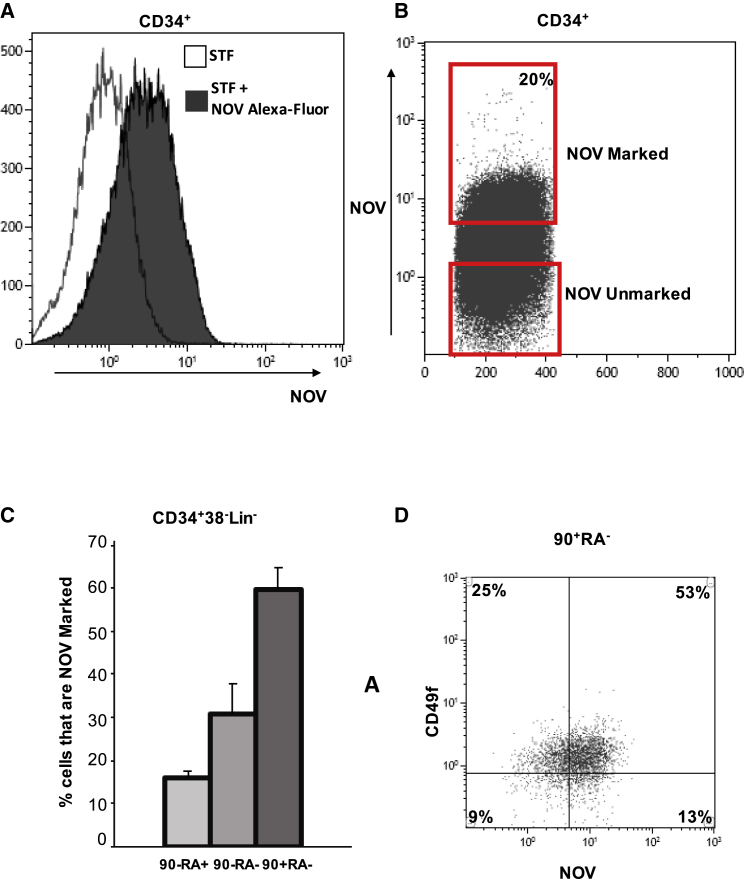
NOV Marks Progenitor Compartments in UCB that Have the Greatest HSC Potential (A) Flow histogram of NOV binding in UCB CD34+ cells incubated for 8 h in SFEM plus either STF or STF + Alexa Fluor 488-NOV. (B) Density plot for cell sorting of NOV-marked and NOV-unmarked CD34+ cells. (C) Percentages of NOV-marked cells in the 90−RA+, 90−RA−, and 90+RA− fractions of the UCB CD34+38−lin− compartment. CD34+ cells were marked with NOV for 8 h before antibody staining; mean + SEM; n = 3. (D) Density plot showing co-segregation of NOV-marked and CD49f+ cells in the UCB CD34+38−90+45RA+Lin− compartment.

We used fluorescently tagged NOV to identify the sub-compartments to which it binds (Doulatov et al., 2012). Recombinant NOV protein was purified from 293T culture supernatant, labeled with an Alexa Fluor dye, and added to freshly isolated UCB CD34+ cells cultured in serum-free expansion medium (SFEM) together with standard cytokines (STF), which are required to maintain cell viability. Approximately 20% of all CD34+ cells were labeled, and maximum staining was achieved after 8 h at 37°C (“NOV-marked” cells; Figures 1A and 1B).

NOV-marked cells were proportionally more abundant in those CD34+ compartments with lower expression of CD38 (Figure S1A). Within the CD34+38-45RA−Lineage-90+ sub-compartment (90+RA−; Figure 1C), which is enriched in progenitors capable of serial transplantation in xenograft recipients, 60% of cells were marked by NOV. Approximately 80% of NOV-marked 90+RA− cells were also CD49f+ (Figure 1D); co-expression of CD49f further enriches for progenitors with the highest capacity for serial transplantation (Notta et al., 2011). Incubation of freshly isolated UCB CD34+ cells in STF alone or NOV+STF altered neither the total number of viable CD34+ cells (not shown) nor the fractions of CD34+ cells that were 90+RA− and 90−RA− (Figures S1B and S1C).

### NOV Increases the Frequency of Functional 90+RA− Cells in Single UCB Units

**Figure 2:**
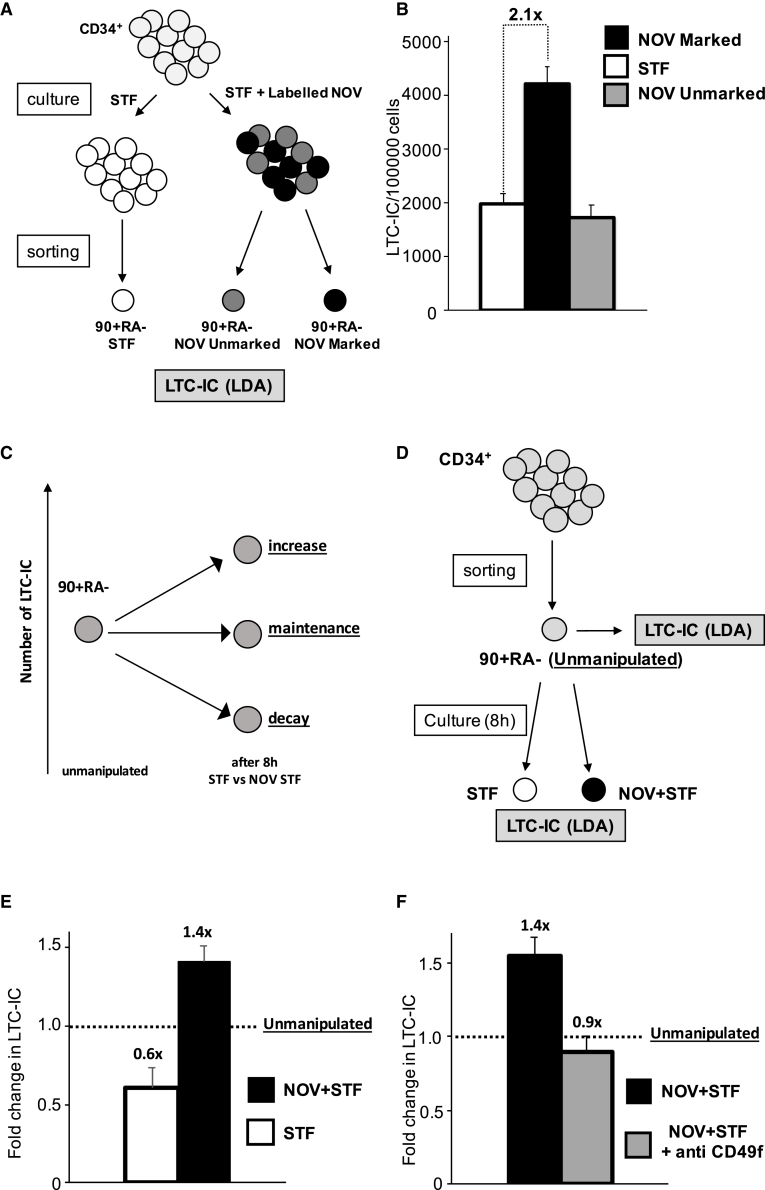
NOV Increases the Frequency of Functional 90+RA− Cells in Single UCB Units (A) Strategy for isolating NOV-marked, NOV-unmarked, and STF-only 90+RA− cells. CD34+ cells are incubated with labeled NOV plus STF or STF alone before antibody staining. (B) LTC-IC frequencies of STF-control, NOV-marked, and NOV-unmarked 90+RA− cells. n = 8 individual UCB unit;, p (STF versus NOV-marked) = 0.0002 (t test); mean + SEM p value by ELDA (http://bioinf.wehi.edu.au/software/elda/; Hu and Smyth, 2009). (C) Possible impacts on LTC-IC number of *ex vivo* culture of 90+RA− cells. Relative to unmanipulated cells (left), the total number of LTC-ICs could decay, be maintained, or increase after 8 h. These may be distinguished by directly comparing LTC-IC frequencies of unmanipulated and cultured cells from the same UCB unit. (D) Strategy to distinguish recruitment and rescue models by enumeration of absolute numbers of LTC-ICs in 90+RA− cells isolated from a single UCB unit before and after exposure to STF or NOV+STF. The absolute number of LTC-ICs present at the start of each culture is the product of the number of unmanipulated cells inoculated and the LTC-IC frequency in unmanipulated cells, absolute numbers at the end calculated from the relevant cell count, and LTC-IC frequency. (E) Fold changes in LTC-IC numbers in STF- and NOV+STF-treated 90+RA− cells. Number of LTC-ICs in the unmanipulated cells inoculated is normalized to 1.0 (dashed line); the fold change in the absolute number of LTC-ICs is calculated relative to this. n = 5 UCB units; mean + SEM; p = 0.006. (F) Fold change in absolute number of LTC-ICs in NOV+STF-treated 90+RA− cells ± anti-CD49f. n = 3; mean + SEM; p (NOV+STF + anti-CD49f versus NOV+STF) = 0.07.

We next asked whether the proportion of functional progenitors in the 90+RA− compartment changed after labeling with NOV. Progenitor frequency was quantified using long-term culture-initiating cell (LTC-IC) assays, which, coupled with limiting dilution assays (LDAs), afford an *in vitro* measure of the clonogenicity of primitive hematopoietic progenitors (Conneally et al., 1997). As outlined in Figure 2A, freshly isolated CD34+ cells were first incubated in either STF or STF plus labeled NOV and the 90+RA− compartments isolated before inoculation into LTC-IC cultures. The LTC-IC frequency was 2.1-fold higher in NOV-marked 90+RA− cells compared to cells that had been exposed but did not bind it (90+RA− NOV-unmarked; Figure 2B) or that were cultured in STF only (90+RA− STF; Figure 2B).

We next asked whether NOV *genuinely* increases LTC-IC number or merely rescues a decay in the capacity to score in LTC-IC assays occurring during *ex vivo* culture in STF. These alternatives are represented in Figure 2C. We compared LTC-IC frequency in NOV and STF-treated 90+RA− cells with that in unmanipulated cells isolated from the same UCB unit using the schema in Figure 2D. The soluble NOV used in this and all subsequent experiments was purchased from R&D Systems (see STAR Methods). We observed that 90+RA− cells did indeed lose LTC-IC activity when cultured in STF (Figure 2E). However, addition of NOV not only compensated this loss but increased the absolute number of LTC-ICs above baseline.

### NOV Activity in 90+RA− Cells Is Mediated by CD49f

In non-hematopoietic cells, NOV binds to and signals through several different integrins, including CD49f (Brigstock, 2003). Because the majority of NOV-marked cells are CD49f+, we asked whether the increase in LTC-ICs seen after exposure to NOV was mediated through this integrin. Again, using the approach described in Figure 2D, we compared the effect of NOV on 90+RA− cells from three individual UCB units in the presence and absence of an unconjugated anti-CD49f antibody. We found that the impact of NOV was abrogated by anti-CD49f (Figure 2F), suggesting that NOV increases LTC-ICs by a CD49f-mediated mechanism in 90+RA− cells and has no impact on the LTC-IC readout of CD49f− cells in this compartment. We postulated that anti-CD49f competes with NOV for binding to CD49f, and consistent with this, in a CD49f+ hematopoietic cell line, pre-incubation with anti-CD49f blocks subsequent binding of Alexa-Fluor-labeled NOV (Figure S1D). Interestingly, incubation of 90+RA− cells with anti-CD49f alone (plus STF) preserved LTC-IC number compared to unmanipulated cells, suggesting that the antibody itself has intrinsic functional activity and is a partial agonist of NOV in this assay (Figure S1E). Consistent with this, we were unable to clearly demonstrate any impact of NOV on prospectively isolated 90+RA−49f+ cells (not shown); we concluded that residual anti-CD49f antibody on the flow-sorted cells blocked the action of NOV.

### NOV Directly Recruits 90+RA− Cells to Score as LTC-ICs

**Figure 3:**
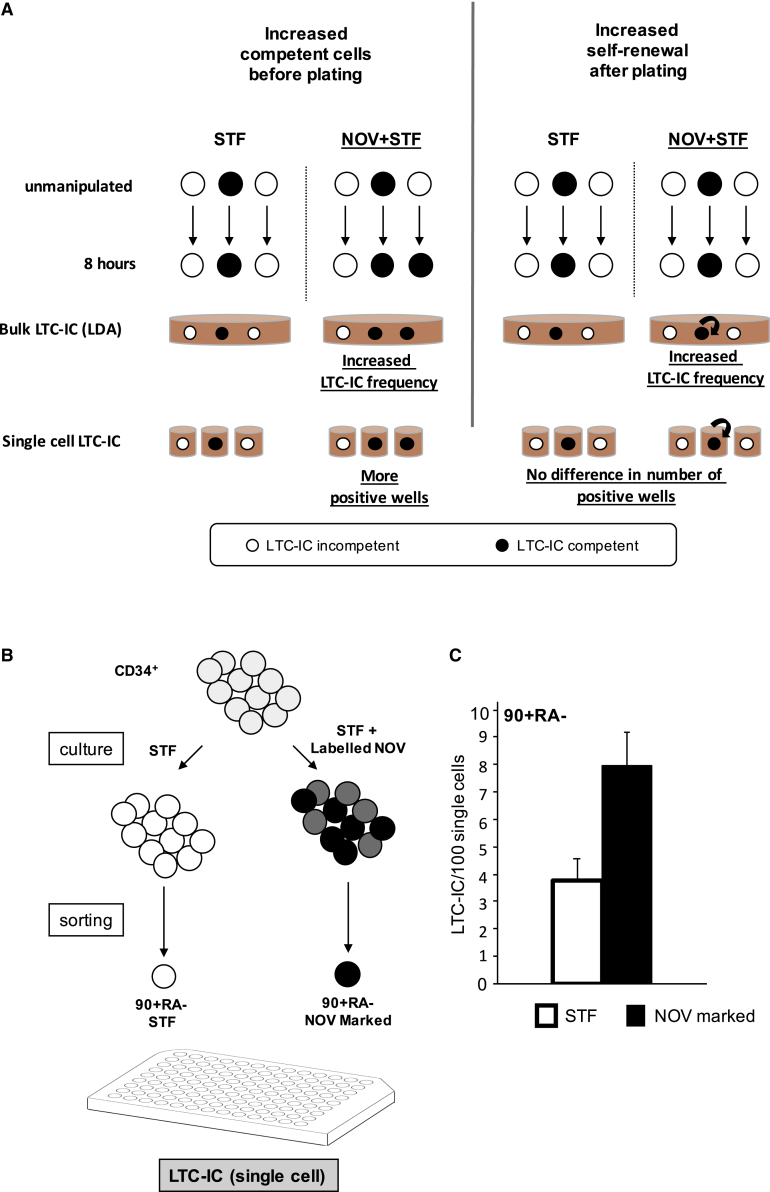
NOV Directly Recruits 90+RA− Cells to Score as LTC-ICs (A) Alternative mechanisms for increased LTC-IC numbers with NOV. Either there are more competent cells before plating (left) or NOV-treated cells undergo more self-renewal after plating (right). These alternatives are indistinguishable in bulk LTC-ICs. In single-cell LTC-ICs, NOV-treated cells generate more positive wells only if there are more LTC-IC competent cells at time of plating, but not if NOV enhanced self-renewal after plating. (B) Strategy to distinguish the two mechanisms. CD34+ cells are incubated with STF alone or labeled NOV+STF prior to single-cell sorting of 90+RA− cells (96-well format). (C) LTC-IC frequency by single-cell deposition in STF-treated and NOV-marked 90+RA− cells (see Figure S2A). 1,056 cells; n = 4 UCB units; mean + SEM;, p (STF versus NOV+STF) = 0.008 (t test).

The increased LTC-IC readout in NOV-treated cultures quantified in the bulk LDAs could arise either because there are more cells capable of scoring in the assay at the end of the 8-h incubation period or because NOV-treated cells undergo more self-renewal after inoculation into LTC-IC cultures (Figure 3A). We therefore assessed the potential of individual prospectively isolated NOV-marked or STIF-treated 90+RA− cells in single-cell LTC-IC assays. This approach affords additional insight because each plated 90+RA− cell and its clonal progeny are contained within the same well, and the well is simply scored as positive or negative. Thus, if the number of positive wells in a single-cell LTC-IC assay increases, then 8-h culture with NOV must have increased the number of LTC-ICs present at the time of plating (Figure 3A). In principle, the number of LTC-IC competent cells in NOV cultures could also be increased by self-renewal during the 8-h incubation prior to inoculation into LTC-IC assays. This is unlikely, however, because we saw no numerical increase in 90+RA− cells (Figure S1B), and furthermore, the mean time to first division of prospectively isolated CD49f+ 90+RA− HSCs in STF is reported to be approximately 80 h (Laurenti et al., 2015).

Using the strategy outlined in Figure 3B, 1,056 90+RA− cells isolated from 4 separate UCB units were analyzed in single-cell LTC-IC assays. NOV-marked cells gave on average 7.98 LTC-ICs (positive wells) per 100 single cells plated versus 3.79 LTC-ICs per 100 single STF-treated cells (Figures 3C and S2A). This 2-fold difference is similar to that seen in our bulk culture LDA (Figures 2B and 2E). Our results indicate that NOV recruits 90+RA− cells that would not otherwise score as LTC-ICs to do so, rather than increasing self-renewal.

### NOV-Recruited HSPCs Do Not Divide during Extended Culture but Retain Their LTC-IC Potential

**Figure 4:**
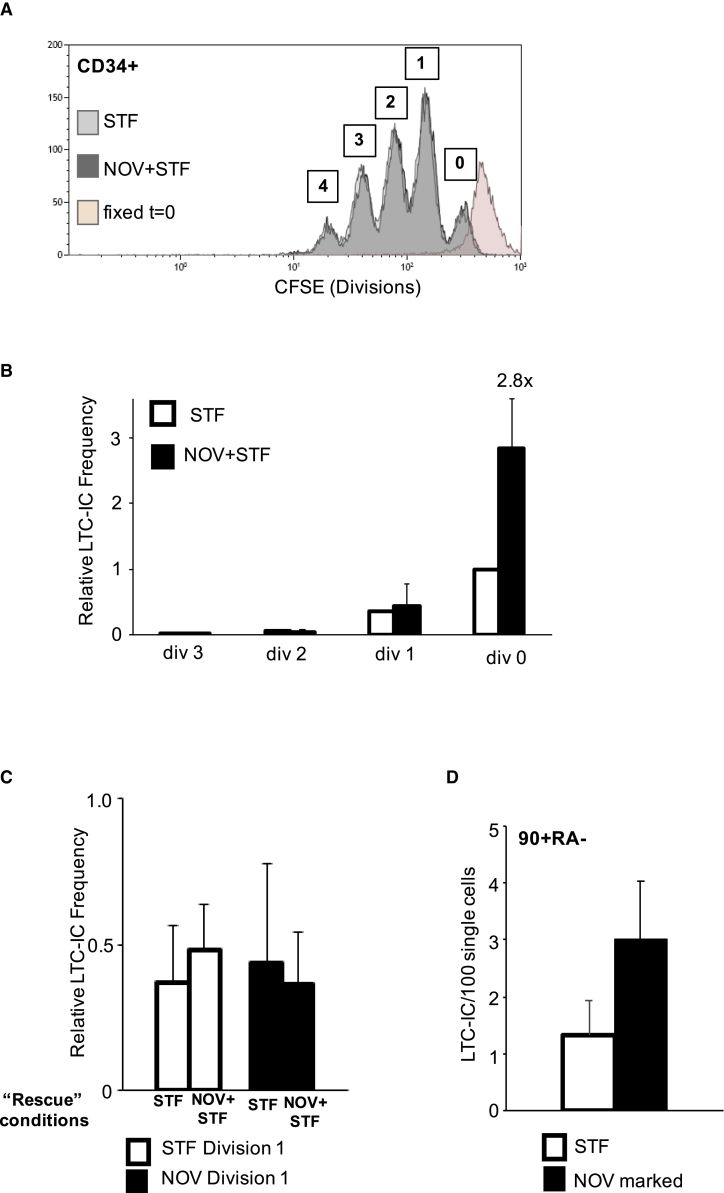
NOV-Recruited HSPCs Do Not Divide in Stem Cell Expansion Culture but Retain Their LTC-IC Potential (A) Representative flow cytometry histogram of CFSE in UCB CD34+ cells in STF or NOV+STF. 0, 1, 2, 3, and 4 divisions are indicated. Pink trace, cells fixed directly after CFSE labeling. (B) Relative LTC-IC frequencies in cells that have undergone 0, 1, 2, or 3 divisions after 6 days’ culture. LTC-IC frequency in div 0 cells in STF is normalized to 1; frequencies at all other divisions under both conditions are calculated relative to this. n = 3; mean + SEM; p (STF versus NOV+STF div 0) = 0.014; p (div1, 2, and 3) = NS. (C) Relative LTC-IC frequencies of div 1 cells from STF or NOV+STF cultures after 8-h “rescue” in either STF or NOV+STF. n = 3; mean + SEM; p = NS. (D) LTC-IC frequency in the progeny of single STF-treated and NOV-marked 90+RA− cells after 14 days’ expansion (see also Figure S2B). NOV-marked cells 3.00 LTC-IC (positive wells) per 100 single cells plated; STF-treated 1.33. 1,056 single cells; n = 4 separate UCB units; mean + SEM; p (STF versus NOV+STF) = 0.04).

We next asked whether NOV-recruited HSPCs divide in extended cultures and, if they do, whether the impact of the NOV signal on LTC-IC activity persists through division. Freshly isolated UCB CD34+ cells were labeled with carboxyfluorescein succinimidyl ester (CFSE) and inoculated into cultures containing STF ± NOV in SFEM. After 6 days, the distribution of CFSE fluorescence was analyzed (Figure 4A). There was no difference in the total number of cells generated in the two culture conditions. We also found no significant difference in the numbers of undivided and divided cells, indicating that NOV did not impact the time to first or successive cell divisions, nor was there any difference in the fraction of CD34+ cells in each division (Figure S3A).

Undivided cells and cells that had undergone one, two, and three divisions were sorted on the basis of CFSE intensity and the LTC-IC frequency determined by LDA. As expected, in both STF and NOV cultures, the LTC-IC frequency was highest in undivided cells (div. 0; Figure 4B), falling with each successive division (Bennaceur-Griscelli et al., 2001). Strikingly, however, NOV treatment resulted in a 2.88× higher LTC-IC frequency in undivided cells compared to STF (Figure 4B). There was no difference in the fraction of undivided cells expressing CD49f (Figure S3B), indicating that the increase in LTC-IC activity is not related to increased CD49f expression.

LTC-IC frequencies in cells that had undergone one or more divisions were unaltered by NOV. Furthermore, isolated div. 1 cells from both STF and NOV+STF cultures did not respond if subsequently treated with NOV+STF for 8 h prior to plating in LTC-IC (Figure 4C), indicating that NOV responsiveness is lost following cell division *ex vivo*. It was not feasible to perform this CFSE experiment with purified 90+RA− cells due to the number of cells required. However, because 90+RA− HSPCs have the highest capacity to score as LTC-ICs and the longest times to first division, we conclude that NOV most likely increases the LTC-IC output in these extended cultures of CD34+ cells by increasing the clonogenicity of 90+RA− HSPCs within the undivided cell population.

In summary, our data show that (1) NOV preferentially binds to a cell compartment enriched in true LT-HSCs as defined by immunophenotype, (2) NOV recruits otherwise non-functional cells within this compartment to score as LTC-ICs, (3) this results in an increase in the absolute number of clonogenic progenitors present in an individual UCB unit, and (4) this increased function is achieved without cell division.

We next asked whether the effect of NOV persists through extended cultures, which might facilitate its use in combination with various HSC expansion strategies (Boitano et al., 2010, Delaney et al., 2010, Fares et al., 2014, Guo et al., 2018). NOV-marked and STF-treated 90+RA− cells were sorted as single cells into medium containing STF only and incubated for 14 days, after which the contents of each well were transferred into LTC-IC conditions. We analyzed 1,056 single 90+RA− cells from four separate UCB units in each condition (Figures S2B and 4D). NOV-marked cells gave 2.25 times as many LTC-ICs in this assay—similar to the increase seen when they were sorted directly into LTC-IC cultures (Figure 3C). This suggests that NOV-activated cells can retain LT-HSC activity over a time frame compatible with current *ex vivo* expansion protocols designed for clinical use.

### NOV-Primed HSPCs Adopt a Distinct Cell State

We next sought insight into the mechanisms underlying the increased LTC-IC activity induced by NOV. Only those NOV-primed CD34+ cells that have not divided exhibit increased clonogenicity. Reduced cell cycling is associated with the greatest capacity for serial transplantation in HSCs (Laurenti et al., 2015) and the highest cloning efficiency *in vitro* in more mature progenitor compartments (Hao et al., 1996). Because HSC quiescence is associated with low levels of both reactive oxygen species (ROS) and c-Myc protein (Cabezas-Wallscheid et al., 2017 and references therein), we first asked whether either is affected by NOV.

**Figure 5:**
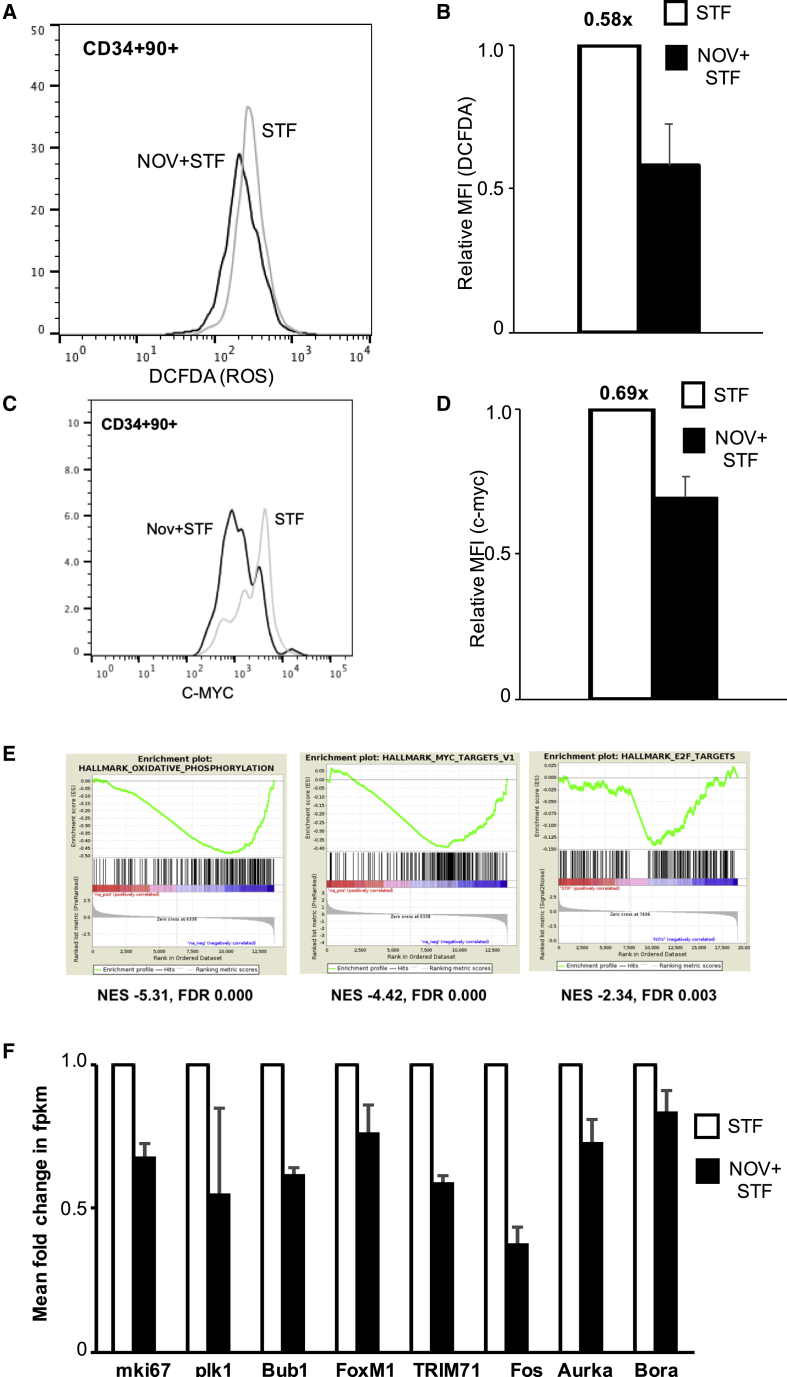
NOV-Primed HSPCs Adopt a Distinct Cell State (A and B) Histogram of fluorescence (A) and median fluorescence intensity (MFI) (B) of DCFDA in 90+RA− cells in STF or NOV+STF; n = 4; mean + SEM; p (STF versus NOV+STF) = 0.062 (t test). (C and D) Flow cytometry histogram (C) and MFI (D) of c-Myc staining in CD34+90+ cells in either STF or NOV+STF. n = 4; mean + SEM; p (STF versus NOV+STF) = 0.02. (E) GSEA of RNA-seq profiles of 90+RA− cells cultured in either STF or NOV+STF, showing reduced transcripts associated with oxidative phosphorylation and MYC and E2F targets (p = 0.00). FDR, false discovery rate; NES, normalized enrichment score. (F) Expression by RNA-seq of indicated genes in 90+RA− cells cultured in either STF or NOV+STF. The fpkm value for STF-treated cells is normalized to 1.0; n = 2.

Total cellular ROS were quantified in 90+RA− cells by flow cytometry after culture for 8 h in STF+/− NOV and staining with 2′,7′-dichlorodihydro fluorescein diacetate (DCFDA). The DCFDA signal in NOV-treated cells was on average 40% lower than in those exposed to STF alone (Figures 5A and 5B), implying lower levels of ROS. We also observed a slight reduction in mitochondrial ROS, which was not statistically significant (Figure S3C). Intracellular c-MYC protein levels were also measured by flow cytometry. UCB CD34+ cells were cultured in either STF ± NOV for 24 h before staining with anti-CD90 and anti-c-MYC antibodies. The level of c-MYC protein was on average 30% lower in NOV-treated CD34+90+ cells compared to STF (Figures 5C and 5D). A similar analysis showed no difference in levels of N-MYC protein (Figure S3D). To test whether the ROS low cells were also c-MYC low, we used CellROX Green, a ROS reporter that withstands cell fixation and permeabilization, allowing co-staining with antibodies to intracellular proteins. We found approximately twice as many ROS low/c-MYC low CD34+90+ cells in the NOV cultures compared to STF controls (Figure S3E).

We then performed RNA sequencing (RNA-seq) on prospectively isolated 90+RA− cells cultured for 8 h in STF ± NOV. Even at this very early time point, gene set enrichment analysis (GSEA) reveals reduced expression of genes encoding proteins associated with oxidative phosphorylation and of c-Myc targets in response to NOV (Figure 5E). Transcription of E2F targets is also reduced (Figure 5E), as are mRNAs of several genes encoding proteins involved in cell cycle entry (Figure 5F), although various gene sets previously associated with quiescence are not enriched (Figure S4A). HSCs have increased glycolytic activity (Suda et al., 2011, Liu et al., 2015), and enhancing glycolysis has been reported to afford UCB expansion (Guo et al., 2018), but initial unbiased analysis showed no enrichment of transcripts associated with glycolysis (Figure S4B). However, several genes encoding glycolytic enzymes are rapidly upregulated in NOV-treated 90+RA− cells (Figure S4C). We quantified expression of one of these enzymes, hexokinase II (HK2), by fluorescence microscopy in 90+RA− cells. The number of HK2-expressing cells was unchanged (Figure S4D), but the levels of HK2 protein were significantly higher with NOV (Figure S4E). Interestingly, HK2 was present in the nucleus under both conditions, but NOV increased the amount of nuclear HK2 (Figure S4F). Nuclear localization of HK2 has been described in human cell lines (Neary and Pastorino, 2010) and also in both UCB-derived HSCs and leukemic stem cells, in which it is associated with increased stem cell activity (G. Thomas et al., 2018, Am. Soc. Hematol., abstract).

### NOV Rapidly Increases the Number of Transplantable HSCs in UCB

These transcriptional data are consistent with our observations that NOV-primed HSPCs do not divide when cultured *ex vivo* and that they have reduced ROS and c-MYC protein. They also suggest that NOV rapidly induces a unique cell state within the quiescent 90+RA− compartment evidenced by changes in glycolytic enzymes, which we have shown leads to increased clonogenicity. However, the most stringent test for HSC activity is serial transplantation in xenograft models, and using the non-obese diabetic (NOD)/severe combined immunodeficiency (SCID) (gamma null) (NSG) xenograft model, we asked whether NOV impacts LT-HSC function *in vivo*.

**Figure 6:**
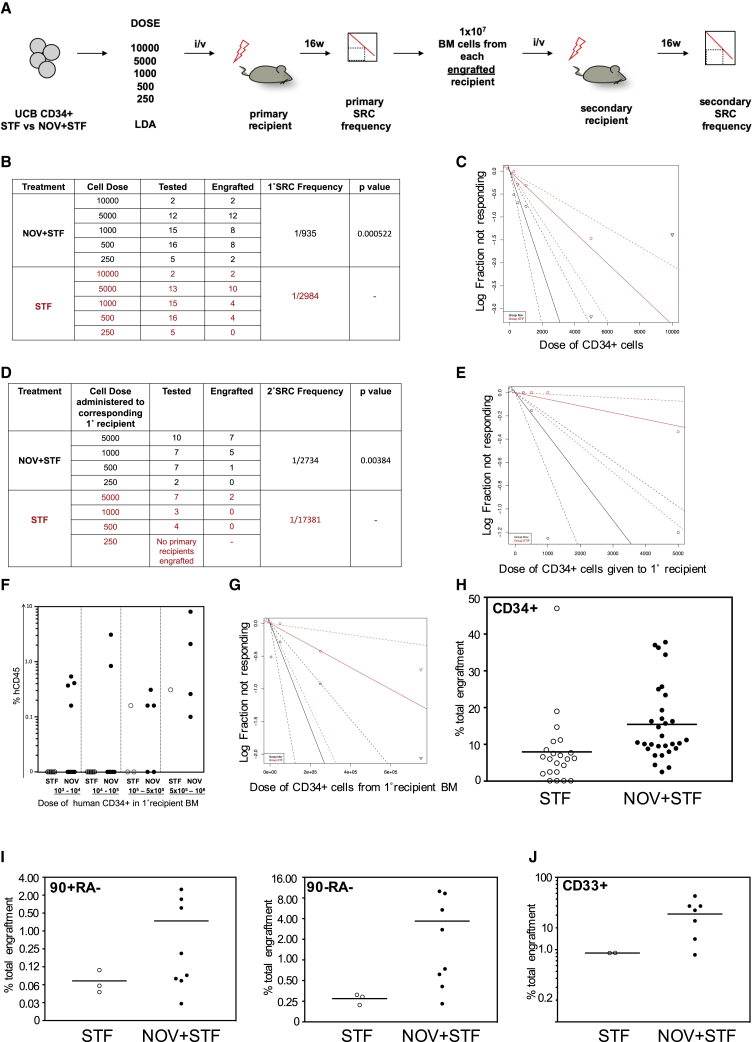
NOV Rapidly Increases the Number of Transplantable HSCs in UCB (A) Strategy for quantifying 1° and 2° SRC frequencies in STF- and NOV-treated CD34+ cells. (B and C) Table summarizing 1° engraftment (B) and graph of 1° SRC frequencies (C) 16 weeks after transplantation of UCB CD34+ cells cultured in either STF or NOV+STF for 8 h. Sold lines indicate best-fit linear model and dashed lines confidence intervals. (D and E) Table summarizing results of 2° assays (D) and graph of 2° SRC frequencies (E) 16 weeks after transplantation of total BM from 1° recipients. 2° SRC frequencies are in terms of the dose of CD34+ cells administered to the corresponding 1° recipient. (F) Level of human reconstitution in 2° recipients at indicated doses of CD34+ cells from 1° BM. (G) Graph of 2° SRC frequencies in terms of dose of CD34+ cells from 1° BM; p = 0.002. See also Figure S5E. (H) CD34+ engraftment in 1° recipients. Mean STF = 7.95%; NOV+STF = 15.43%; p = 0.011 (t test). (I) 90+RA− (left) and 90−RA− engraftment. Mean 90+RA− STF = 0.072%, NOV+STF = 0.74%, p = 0.09; 90−RA− STF = 0.27%, NOV+STF = 3.68%, p = 0.05 (t test). (J) Myeloid (CD33+) engraftment in secondary recipients. Mean STF = 8.98%; NOV+STF = 31.08%; p = 0.011 (t test).

Freshly isolated UCB CD34+ cells were cultured in either STF ± NOV for 8 h and doses of between 250 and 10,000 cells transplanted intravenously into sub-lethally irradiated NSG recipients. At 16 weeks, bone marrow (BM) was harvested and the frequency of primary SCID repopulating cells (1° SRC) determined. 1 × 10^7^ harvested BM cells from primary recipients with demonstrable human engraftment were transplanted into secondary NSG recipients. After a further 16 weeks, engraftment was analyzed and the 2° SRC frequency scored in terms of the dose of cells given to the corresponding primary recipients (Figure 6A). Mice in which human CD45+ cells accounted for at least 0.1% of total BM hematopoietic cellularity, and in which the human cells expressed at least two of CD33, CD235, or CD19 surface markers, were considered to show engraftment.

We found NOV treatment increases the frequencies of both 1° and 2° SRCs in UCB. 32/50 1° recipients of NOV-treated cells showed human engraftment, compared with 20/51 STF recipients (Figure 6B), and LDA demonstrated the 1° SRC frequency in NOV-treated CD34+ cells to be 1/935, compared with 1/2,984 in STF-treated cells (Figures 6B and 6C). At each dose of input cells, primary recipients of NOV- and STF-treated UCB showed similar levels of human reconstitution—although at lower doses, more recipients of NOV-treated cells showed engraftment (Figure S5A). There was no lineage bias in primary recipients; the levels of human myeloid and B-lymphoid cells were similar in all recipients with human reconstitution (Figures S5B–S5D).

Secondary engraftment was seen in 13/26 recipients of BM from NOV primary recipients and 2/14 from STF primary recipients, and the 2° SRC frequency in NOV-treated CD34+ cells was 1/2,734, compared with 1/17,381 for STF-treated (Figures 6D and 6E). The level of human engraftment in secondary recipients was in the range 0.1%–8.1% (Figure 6F), with a minimum of 10^6^ total events collected. When calculated in terms of the estimated number of human CD34+ cells administered from the BM primary recipients, 2° SRC frequencies were 1/126,382 for NOV-treated cells and 1/594,733 for STF treated (Figures 6G and S5E). This 5- to 6-fold increase in the frequency of HSCs capable of secondary engraftment demonstrates that an 8-h pulse of NOV enhances durable long-term engraftment, signposting its potential clinical benefits in UCB transplantation.

### The Proportion of 90+RA− Cells Is Increased in Primary Recipients of NOV-Treated UCB

This key finding of increased secondary engraftment indicates the human grafts in primary recipients of NOV-treated UCB contain more functional HSCs. This could be either because the overall level of human reconstitution is greater in recipients of NOV-treated cells (Notta et al., 2010), because the level of human reconstitution is the same but the grafts in NOV recipients contain proportionally more phenotypic HSCs, or both.

Although 1° recipients of NOV- and STF-treated UCB showed similar levels of human reconstitution (Figure S5A), the percentage of CD34+ cells in the human grafts of NOV recipients was significantly higher (Figure 6H). Where possible, we also quantified 90+RA− and 90−RA− cells and found that the 90+RA− compartment was 10× larger in the recipients of NOV-treated HSPCs (0.74% versus 0.072%) and the 90−RA− compartment was 13× larger (3.68% versus 0.27%; Figure 6I). Thus, primary recipients of NOV-treated cells show the same overall level of human reconstitution as STF recipients, but the proportion of phenotypic HSCs within the grafts is higher. This is consistent with *ex vivo* recruitment of HSCs by NOV, although we cannot exclude that NOV-treated HSCs may also show increased self-renewal *in vivo* after lodging in the BM.

We also found that the fraction of human myeloid cells was higher in the engrafted secondary recipients of NOV-treated UCB (Figure 6J). Sustained myeloid engraftment through secondary transplantation further supports our conclusion that the increased secondary transplantation is due to true HSC activity rather than the persistence of a primitive B cell progenitor present in the primary recipients.

We next assessed the impact of NOV on ST-HSCs. ST-HSCs are responsible for early hematopoietic reconstitution, which is delayed in patients receiving UCB transplants, causing considerable morbidity. UCB CD34+ cells were cultured in either STF ± NOV and transplanted intravenously at a saturating dose (10,000 cells). Bone marrow reconstitution was assessed after 8 weeks. The overall levels of human reconstitution were similar, indicating that the recruitment of LT-HSCs by NOV is not at the expense of ST-HSCs and less primitive progenitors (Figure S6A) and that the kinetics of overall early engraftment are not significantly altered. Strikingly, at this relatively early time point, recipients of NOV-treated cells showed higher levels of CD34+38lo90+ hematopoietic progenitors (0.33% versus 0.06%; Figure S6B), consistent with our results at 16 weeks. Furthermore, CD15+ myeloid cells, which include mature granulocytes and monocytes, were modestly but significantly higher in the recipients of NOV-treated cells (2.86% versus 1.12%; Figure S6C), although there was no difference in either total myeloid, B lymphoid, or erythroid reconstitution (Figure S6D).

### NOV Enhances Engraftment by a Single UCB Unit

Finally, we directly tested whether an 8-h treatment with NOV increases the number of 1° SRCs in a single UCB unit compared to unmanipulated cells from the same unit. Formal demonstration of this would support the clinical use of NOV: the therapeutic potential of any *ex vivo* manipulation of UCB hinges on direct and unambiguous evidence that treated cells perform better than unmanipulated cells from the same unit in xenograft assays.

**Figure 7:**
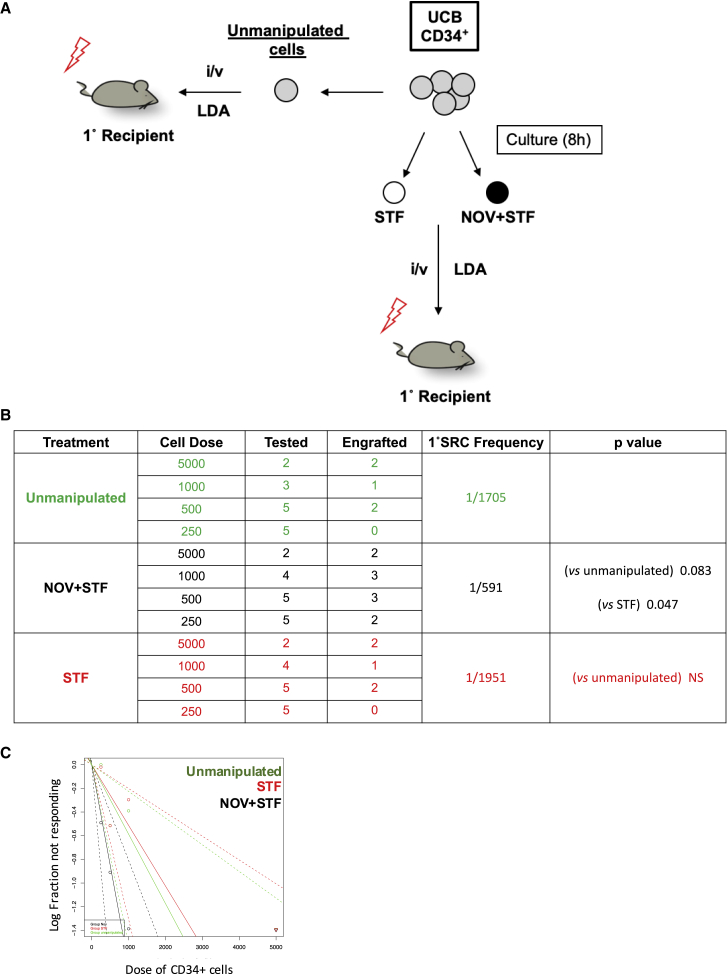
NOV Enhances Engraftment by a Single UCB Unit Experimental strategy (A), table summarizing 1° engraftment (B), and graph of 1° SRC frequencies (C) in a single UCB unit before (unmanipulated cells, green) and after incubation with either STF (red) or NOV+STF (black).

UCB CD34+ cells from a single donor were either transplanted immediately in a LDA, with cell doses ranging from 250 to 5,000 *per* mouse (“unmanipulated cells”; Figure 7A) or cultured in either STF ± NOV for 8 h before transplantation (again by limiting dilution). We assessed bone marrow reconstitution after 16 weeks (Figures 7B and 7C) and found that the 1° SRC frequency in NOV-treated cells was 1/591, approximately 3-fold higher than in either unmanipulated cells (1/1,705) or STF-treated cells (1/1,951). This result supports both the clinical potential of NOV and our hypothesis that it acts in compartments that are enriched in HSCs by recruiting cells that would not otherwise score in transplantation assays to do so.

## Discussion

Our key observations are that short exposure to NOV increases the number of UCB cells capable of serial engraftment in xenograft recipients and that, when CD34+ cells from a single donation are tested before and after treatment with NOV, the absolute number of engraftable cells is increased. This occurs without increases *ex vivo* in either the total number of CD34+ cells or the fraction of phenotypic (90+RA− or 90−RA) HSCs and is not at the expense of engraftment by ST progenitors. Taken together with our mechanistic experiments, we postulate that this occurs through a recruitment rather than an expansion mechanism.

Within the CD34+ compartment, there are many different cell types, and the exact identity of the target cell for many stem cell expansion agents is unclear. Similar caveats apply to our study; however, our use of fluorescently tagged NOV protein shows that NOV binds a broad range of HSPCs (60% of 90+RA− and 30% of 90−RA− cells), that 80% of NOV-marked 90+RA− cells are also CD49f+, and that CD49f antibodies abrogate binding of NOV and block its effect. The simplest interpretation of our findings is that NOV targets phenotypic LT-HSCs. In accordance with this, the greatest functional impact of NOV is on the number of cells capable of secondary engraftment (a 6.4-fold increase in 2° SRC versus 3.2-fold for 1° SRC). Furthermore, the key features of engraftment in recipients of NOV-treated UCB mirror those observed when engraftment by prospectively isolated 90+RA− cells—which are relatively enriched in LT-HSCs—is compared with engraftment by 90−RA− cells (Majeti et al., 2007), namely (1) increased numbers of mice showing engraftment with limiting doses of human cells, (2) no increase in the average level of human chimerism, and (3) increased levels of human CD34+ cells but (4) no alteration in the mature lineage composition of the human graft.

In the absence of increased numbers of phenotypic LT-HSCs, the enhanced serial transplantation seen after NOV treatment could be due to either improved homing of LT-HSCs to the BM, their enhanced survival, or to their functional activation prior to transplantation. Our LTC-IC studies, where homing is not involved, suggest that NOV functionally activates phenotypic HSCs, recruiting them to score in transplantation assays. This is highlighted in the single-cell experiments, in which NOV increased the number of positive wells scored. Further evidence that NOV does not act through self-renewal comes from our CFSE experiments with CD34+ cells, where we show that the NOV-responsive cells lie in the undivided fraction.

The delayed cell cycle entry reported in prospectively isolated LT-HSCs (Laurenti et al., 2015) further supports our conclusion that NOV is priming LT-HSCs to score rather than increasing their self-renewal *ex vivo*. Our mechanistic studies explored the effect of NOV during the 8-h *ex vivo* incubation period and the nature of the manipulated product that might be used clinically. *In vivo*, the consequence of NOV treatment is enhanced engraftment, but the precise history of individual NOV-treated HSCs after transplantation is unknown.

NOV-treated 90+RA− cells exhibit low ROS, downregulation of c-*myc* and its targets, reduced expression of genes involved in oxidative phosphorylation, and reduced E2F-dependent transcripts. Each has been reported in HSCs showing the greatest capacity for transplantation. Identifying the role played by individual components within HSCs, although challenging (Nakamura-Ishizu et al., 2014), has been addressed largely through elegant genetic approaches in mice (Cabezas-Wallscheid et al., 2017) but remains technically difficult in the human setting. Stem cells are increasingly recognized as being highly heterogeneous in terms of their functional state (Haas et al., 2018, Knapp et al., 2018). Thus, it is likely that multiple physiological changes may be required to converge in a single HSC in order to achieve a change in state or functional output. Interestingly, we observed a range of seemingly disparate changes in NOV-treated cells; despite their low levels of ROS and c-MYC—features associated with quiescent stem cells—we observed increased transcription of genes encoding glycolytic enzymes, increased amounts of nuclear hexokinase II protein, and increased transcription of genes encoding ribosomal proteins (not shown).

We have explored the effect of soluble NOV on normal HSCs, and our experiments with a blocking antibody suggest it signals through CD49f. In other systems, soluble NOV is internalized and can accumulate in diverse cellular locations, including both the cytoplasm and the nucleus, although its role in these locations is poorly understood (Sin et al., 2009). It remains possible that, besides CD49f signaling, soluble NOV may mediate its effects intracellularly. Indeed, we have previously shown that forced expression of NOV in HSCs enhances engraftment in xenograft models (Gupta et al., 2007), but whether this is due to intracellular expression of NOV, secretion and subsequent signaling through surface CD49f, or both is unclear.

It is estimated that 700,000 UCB units have been donated for transplantation (Ballen, 2017) since 1988. Currently, at least 30% of donations are deemed unsuitable due to low cell count (Wang et al., 2014). Guidelines recommend that patients receive one UCB unit, unless a single unit with an adequate cell dose is unavailable (Hough et al., 2016). However, many older patients require two units to ensure adequate early hematopoietic reconstitution, in particular, the recovery of adequate numbers of circulating neutrophils and platelets. As a result, UCB expansion technologies to increase the numbers of available HSCs in donated units have emerged. They are typically evaluated in mouse xenograft assays, which remain the experimental “gold standard” by which ST- and LT-HSC activity is assessed, although the precise clinical correlates of primary and secondary engraftment in mice are unknown. Several small molecules have been developed for UCB expansion, and in essence, they attempt to force HSC divisions *ex vivo* and bias them to self-renewal by inhibiting differentiation. However, expansion cultures favor committed progenitors and ST-HSCs over LT-HSCs, which is perhaps unsurprising given their relative cell-cycle latencies (Laurenti et al., 2015). Although the expansion of ST-HSCs is highly desirable and, in early-phase clinical trials, has yielded improvements in early hematopoietic reconstitution, as yet, no single approach that affords both progenitor expansion and enhanced LT-HSC function from a single UCB unit has emerged (Nikiforow and Ritz, 2016). Interestingly, when NSG recipients are assessed at the earlier time point of 8 weeks, NOV increases engraftment of both mature (CD15+) myeloid cells and phenotypic stem/progenitor cells, although total human engraftment is not obviously changed. This implies a trajectory of early hematopoietic reconstitution that is clinically favorable; the delayed emergence of mature neutrophils is a cause of considerable toxicity in UCB transplantation. It would be interesting to study very early time points, but the mouse xenograft model is limited in this regard, and the ultimate arbiter of medical utility is the demonstration of benefit in a clinical trial.

Using a “cocktail” of proliferative cytokines and HSC-active small molecules together with agents that promote homing may be necessary to maximize the clinical potential of each UCB unit (Broxmeyer, 2016, Patterson and Pelus, 2018). The properties we describe make NOV an excellent candidate for such combinatorial approaches. Its rapid action makes it a good candidate to test in concert with agents that enhance homing (e.g., Popat et al., 2015), and its impact on ROS suggests potential synergy with the processing of UCB under hypoxic conditions, which is known to increase 1° SRC frequency (Mantel et al., 2015). Interestingly, there is evidence in the literature that NOV is positively regulated by hypoxia signaling pathways (e.g., Tran et al., 2013). Furthermore, the persistence of NOV-primed cells in the undivided fraction of LT cultures of CD34+ suggest that it would still enhance LT-HSC activity if used in combination with lengthier conventional HSC expansion protocols.

The therapeutic potential of NOV may extend beyond UCB transplantation; rapid augmentation of HSC activity may also enhance *ex vivo* gene therapy strategies. However, this commonly uses HSCs from non-UCB sources, and so we tested it on mobilized peripheral blood CD34+ cells. In a preliminary study, we observed that, similar to its effects in UCB-derived HSCs, NOV increases LTC-IC frequency in this adult HSC compartment by approximately 3-fold (Figure S6E).

In conclusion, our study identifies rapid recruitment of functional LT-HSCs as a new strategy for enhancing UCB transplantation, which merits further investigation in the clinical setting.

## STAR★Methods

### Key Resources Table

**Table:** 

REAGENT or RESOURCE	SOURCE	IDENTIFIER
**Antibodies**		

Mouse anti human CD45RA BV711	Biolegend	Cat # 304138, RRID:AB_2563815
Mouse anti human CD90 PE	Biolegend	Cat # 328110, RRID:AB_893433
Mouse anti human CD34 APC-Cy7	Biolegend	Cat # 343514, RRID:AB_1877168
Mouse anti human CD38 BV785	Biolegend	Cat # 303530, RRID:AB_2565893
Mouse anti human CD14 Pe-Cy5	eBioscience	Cat # 15-0149-42, RRID:AB_2573058
Mouse anti human CD15 FITC	BD Biosciences	Cat # 347423, RRID:AB_400299
Mouse anti human CD16 Pe-Cy5	Biolegend	Cat # 302010, RRID:AB_314210
Mouse anti human CD56 Pe-Cy5	Biolegend	Cat # 304608, RRID:AB_314450
Mouse anti human CD19 Pe-Cy5	Biolegend	Cat # 302210, RRID:AB_314240
Mouse anti human CD2 Pe-Cy5	Biolegend	Cat # 300210, RRID:AB_314034
Mouse anti human CD3 Pe-Cy5	Biolegend	Cat # 300310, RRID:AB_314046
Mouse anti human CD123 Pe-Cy5	Biolegend	Cat # 306008, RRID:AB_493574
Mouse anti human CD235a Pe-Cy5	BD Biosciences	Cat # 559944, RRID:AB_397387
Rat anti human/mouse CD49f BV650	BD Biosciences	Cat # 567707
Rat anti mouse CD45 FITC	Biolegend	Cat # 103108, RRID:AB_312973
Mouse anti human CD45 AF700	Biolegend	Cat # 304023, RRID:AB_493760
Mouse anti human CD34 FITC	BD Biosciences	Cat # 348053, RRID:AB_2228982
Mouse anti human CD33 PE	BD Biosciences	Cat # 555450, RRID:AB_395843
Mouse anti human CD19 APC	BD Biosciences	Cat # 561742, RRID:AB_10894000
Mouse anti human CD235a APC	BD Biosciences	Cat # 561775, RRID:AB_10894583
Mouse anti human/mouse NMYC AF488	Novus Biologicals	Cat # NB200-109AF488
Mouse anti human c-Myc DL650	Abcam	Cat # Ab117487, RRID:AB_10900341
Rat anti human/mouse CD49f unconjugated	E-Bioscience	Cat # 14-0495-82, RRID:AB_891480
Rabbit anti human/mouse HK II unconjugated	CST	Cat # 2867, RRID:AB_2232946
Donkey ant rabbit IgG AF594	ThermoFisher	Cat # A21207, RRID:AB_141637

**Biological Samples**		

Human Umbilical Cord Blood	Stem Cell Technologies	Cat # 70007
Mobilized Peripheral Blood	Hospital Clínico San Carlos, Madrid	N/A

**Chemicals, Peptides, and Recombinant Proteins**		

Antibiotic antimycotic solution (100x)	SIGMA-ALDRICH	Cat # A5955
Bovine serum Albumin (30% in DPBS)	SIGMA-ALDRICH	Cat # A9576
Chloroform	Fisher Scientific	Cat # 10293850
Dimethylsulfoxide (DMSO)	SIGMA-ALDRICH	Cat # D2650
Distilled water (tissue culture)	GIBCO	Cat # 15230188
DNase 1	Roche	11284932001
Dulbecco’s Modified Eagle Medium (DMEM, high Glucose, Pyruvate)	GIBCO	Cat # 41966029
Dulbecco’s Phosphate Buffered Saline (DPBS)	GIBCO	Cat # 14190094
Ethanol	Fisher Scientific	Cat # 10428671
Fetal Bovine Serum Heat Inactivated	GIBCO	Cat # 10500064
Fugene6 Transfection reagent	PROMEGA	Cat # E2692
Geneticin (G418)	ThermoFisher	Cat # 11811023
Gelatin solution (2%)	SIGMA-ALDRICH	Cat # G1393
GenElute linear polyacrylamide (LPA)	SIGMA-ALDRICH	Cat # 56575
Hydrocortisone	Stem Cell Technologies	Cat # 07904
Hygromycin-B	SIGMA-ALDRICH	Cat # H3274
Hoechst 33258 (10mg/mL solution)	MOLECULAR PROBES	Cat # H3569
Iscove’s Modified Dulbecco’s Medium (IMDM)	GIBCO	Cat # 12440053
Isopropanol	Fisher Scientific	Cat # 10497070
MES (6-Methoxy-3,4-dihydro-2(1*H*)-naphthalenone) sol^n^ (1M)	SIGMA-ALDRICH	Cat # M1317
MethoCult H4435 Enriched	Stem Cell Technologies	Cat # 04435
Myelocult H5100	Stem Cell Technologies	Cat # 05150
Optimem	GIBCO	Cat # 51985026
Paraformaldehyde 16% (w/v) methanol free	VWR	Cat # 0219998380
RBC Lysis Buffer (10x)	Biolegend	Cat # 420301
Recombinant Human Flt3-Ligand	PEPROTECH	Cat # 300-19
Recombinant Human NOV/CCN3	R&DSYTEMS	Cat # 1640-NV-050
Recombinant Human SCF	PEPROTECH	Cat # 300-07
Recombinant Human TPO	PEPROTECH	Cat # 300-18
RPMI 1640 Medium	GIBCO	Cat # 21875034
StemSpan SFEM	Stem Cell Technologies	Cat # 09650
TBS (10x) PH7.4	Severn Biotech Ltd	Cat # 20-7301-10
Triethylamine (7.17M)	SIGMA-ALDRICH	Cat # T0886
Triton X-100	SIGMA-ALDRICH	Cat # T8787
TRIzol	ThermoFisher	Cat # 15596018
Trypsin Solution (10x)	SIGMA-ALDRICH	Cat # 59427C
Ultra-pure DEPC-treated water (RNA work)	ThermoFisher	Cat # 750023
Vectashield antifade mounting medium with DAPI	Vector Laboratories	Cat # H-1200

**Critical Commercial Assays**		

Agencourt AMPureXP magnetic beads	Beckman Coulter	Cat # A63881
Agilent High Sensitivity DNA Kit	Agilent	Cat # 5067-4626
Agilent RNA 6,000 Pico Kit	Agilent	Cat # 5067-1513
Alexa Fluor 488 succinimidyl ester	MOLECULAR PROBES	Cat # A20000
Anti-FLAG M2 affinity gel	SIGMA-ALDRICH	Cat # A2220
CellROX Green	MOLECULAR PROBES	Cat # C10444
Cell Trace CFSE Proliferation Kit for Flow Cytometry	MOLECULAR PROBES	Cat # C34554
CellTrics 30μM sterile cell filter	SYSMEX	Cat # 040042326
Centricon Plus 70 Centrifugal filter	MILLIPORE	Cat # UFC701008
2′,7’-dichlorodihydrofluorescein di-acetate (DCFDA)	MOLECULAR PROBES	Cat # D399
3xFLAG peptide	SIGMA-ALDRICH	Cat # F4799
LS columns	MiltenyiBiotec	Cat # 130-042-401
μ Columns MiltenyiBiotec	MiltenyiBiotec	Cat # 130-042-701
MBC CD34 Microbead kit	MiltenyiBiotec	Cat # 130-091-586
Mitosox Red	Molecular Probes	M36008
Nextera XT DNA Library Preparation Kit	Illumina	Cat # FC-131-1024
Perfix nc no centrifuge intracellular staining kit	BECKMAN COULTER	Cat # B31167
Qubit dsDNA High Sensitivity Assay Kit	Invitrogen	Cat # Q32851
SMARTer Ultra Low Input RNA Kit for Sequencing – v3	Takara Clontech	Cat # 634849
TruSeq SBS v3-HS kit	Illumina	Cat # FC-401-3002

**Deposited Data**		

RNASeq Data	EGA	EGAS00001003979

**Experimental Models: Cell Lines**		

Jurkat human T cell Line	Weatherall Institute of Molecular Medicine, Oxford UK	N/A
293T human fibroblast cell line	Weatherall Institute of Molecular Medicine, Oxford UK	N/A
M210B4 mouse stromal feeder cell line	Stem Cell Technologies	https://cdn.stemcell.com/media/files/pis/29301-PIS_1_0_2.pdf
S1/S1 mouse stromal feeder cell line	Stem Cell Technologies	https://cdn.stemcell.com/media/files/pis/29301-PIS_1_0_2.pdf

**Experimental Models: Organisms/Strains**		

NOD.Cg *Prkdc*scid*Il2rg*tm1Wjl /SzJ	Charles River	https://www.criver.com/user/login?region=3671&destination=/products-services/find-model/jax-nsg-mice%3Fregion%3D3671

**Recombinant DNA**		

pcDNA3-NOV	Gupta et al. 2007	N/A

**Software and Algorithms**		

Extreme Limiting Dilution Analysis (ELDA	Hu and Smyth (2009)http://www.sciencedirect.com/science/article/abs/pii/S0022175909001951	http://bioinf.wehi.edu.au/software/elda/
FlowJo v10	FlowJo, LLC	N/A
FACSDiva	BD	
Graphpad QuickCalcs	GraphPad Software	https://www.graphpad.com/quickcalcs/
GSEA		https://www.gsea-msigdb.org/gsea/index.jsp
Kaluza	Beckman Coulter	N/A
Prism 7	GraphPad Software	N/A
Volocity	Quorum Technologies	N/A
BioConductor version 3.7	https://Bioconductor.org	N/A
TRimGalore	https://github.com/FelixKrueger/TrimGalore	N/A
tophat2	https://ccb.jhu.edu/software/tophat/index.shtml	N/A

### Lead Contact and Materials Availability

Further information and requests for resources and unique/stable reagents should be directed to and are available without restriction from the lead contact responsible for materials availability, Tariq Enver (t.enver@ucl.ac.uk). The study did not generate new unique reagents.

### Experimental Model and Subect Details

#### Umbilical cord blood

Frozen human umbilical cord blood cells (aliquots of 1.5x10^8^ cells from anonymous single donors) were purchased directly from Stem Cell Technologies (Cat # 70007, https://www.stemcell.com/human-cord-blood-mononuclear-cells-frozen.html).

#### Mobilized adult peripheral blood mononuclear cells

These were a kind gift of Dr Eduardo Anguita (Hospital Clínico San Carlos, Madrid Spain), and obtained according to procedures approved by the local Research Ethics Board. All viable cells were stored at −150°C.

#### Cell Lines

Human Jurkat and 293T cell lines were a kind gift of Professor WG Wood, MRC Molecular Haematology Unit, Weatherall Institute of Molecular Medicine, Oxford, UK.

M210B4 and S1/S1 mouse stromal feeder cell lines were purchased from Stem Cell Technologies (https://cdn.stemcell.com/media/files/pis/29301-PIS_1_0_2.pdf)

#### Mice

NOD/SCID IL2Rγ^null^ (NSG, NOD.Cg *Prkdc*scid*Il2rg*tm1Wjl /SzJ) mice were purchased from Charles River. All animal experiments were performed in accordance with United Kingdom Home Office regulations. Mice that were used in xenograft experiments were aged between 8 and 12 weeks, and an equal number of males and females were used.

Animals were housed in individually ventilated cages (IVC) under specific pathogen free (SPF) conditions.

### Method Details

#### Preparation of labeled NOV

The pcDNA3-NOV mammalian expression vector encodes recombinant human NOV with cloned carboxy-terminal FLAG and His_6_ protein tags (described in Gupta et al., 2007). 293T fibroblasts were cultured in DMEM/10% heat inactivated FCS (DMEM 10%) to 70% confluence in four 75cm^2^ flasks, and the medium replaced. 56.6μL Fugene 6 transfection reagent was added to 820μL Optimem, followed by 10.3μg pcDNA3-NOV (concentration 2μg/L). The mixture was allowed to stand at room temperature for 30 minutes and then 220μL added drop wise to each of the 293T flasks. After 12 hours, the medium in each flask was replaced with 12.5mL Fresh DMEM 10%, and after a further 48 hours the medium from the four flasks was pooled and spun at 300 g for 10minutes. The supernatant was transferred to a Centricon Plus 70 Centrifugal filter device, and spun at 2500rpm for 3-4 hours. The concentrated medium (typically 700 – 800 μL from 50mL culture supernatant) was applied to 100μL Anti-FLAG M2 affinity gel, which had been previously washed in TBS according to the manufacturer’s instructions. The total volume of this mixture was the made to 1.1mL with TBS and it was incubated overnight at 4° with continuous rotation. The affinity gel was then washed 5 x in 1mL TBS and incubated at 4°C in 300μL 3xFLAG peptide (0.2μg/L in TBS) with continuous rotation for 40 minutes. The gel was spun at 16000 g for 2 minutes and the supernatant, containing eluted NOV protein, was collected and made up to 1mL with 0.1M Sodium Bicarbonate (pH8.3). 500μg Alexa Fluor 488 succinimidyl ester (reconstituted in DMSO) was then added and the labeling reaction incubated for 1 hour at room temperature, protected from light. At the end of the reaction 50μL anti His-tag microbeads were added, and the mixture incubated on ice for 30 minutes. A μMACS column was inserted into a μMACS separator and primed with 1mL lysis buffer (150mM NaCl, 1% Triton X-100, 50mM Tris pH8). The labeling reaction/microbead mixture was applied to the column, and the column washed twice with 1mL 0.1M Sodium Bicarbonate (pH8.3), followed by two washes with 250μL 20mM Tris HCL (pH 7.5). 20μL of 0.1M Triethylamine (pH 11.8). 0.1% Triton 100 (TE/TX100) was added to to column and incubated for 5 minutes at room temperature, at which point 150μL TE/TX100 was added and the eluate from the column collected in a tube containing 9μL 1M MES. The eluates (containing labeled NOV) were stored as aliquots of 10μL at −20°C.

#### Isolation of CD34+ MNCs from UCB or mobilized PBMNCs

A single UCB or PBMNC unit was thawed at 37°C in a water bath, and the total MNC suspension transferred to a 50mL centrifuge-tube containing 500μL of 1mg/mL DNase 1 (Roche 11284932001, reconstituted in sterile PBS). The cells were resuspended at room temperature, and 19.5mL IMDM/10% Heat inactivated FCS (IMDM 10%) added prior to centrifugation for 5 minutes at 200 g. The supernatant was removed and the cell pellet resuspended in 500μL DNase 1. 19.5mL IMDM 10% was then added followed by centrifugation for 5 minutes at 200 g, after which the supernatant was removed and the pellet resuspended in 500μL of DNase 1 followed by 9.5ml Dulbecco’s Phosphate Buffered Saline (DPBS, GIBCO 14190094)/10% Heat inactivated FCS (PBS 10%). The MNC were enumerated and spun for 5 minutes at 200 g. The supernatant was removed and the cell pellet resuspended in 200μL DNase 1 followed by the appropriate volume of PBS 10% required to achieve a cell concentration of 10^8^/500μL. For every 500μL of resuspended MNCs, 143μL FcR blocking reagent from the Mitenyi MBC CD34 Microbead kit was then added and the suspension mixed by inversion. Then 71.5μL CD34 Microbeads were added *per* 500μL followed by further mixing by inversion, and this suspension was then incubated for 20 minutes at 4-8°C in a standard laboratory refrigerator. At the end of the incubation the cells were washed with 20mL PBS 10%, spun for 5 minutes at 200 g and the cell pellet resuspended in 200μL DNase 1. The total volume was then made up to 10mL with PBS 10%, and the cell suspension passes through a pre-wetted CellTrics 30μM sterile cell filter onto a Miltenyi LS column (primed with 15 mL PBS 10%) in a MidiMACS separator. The column was washed with a further 10mL of PBS 10%, and CD34+ enriched cells eluted in 5mL PBS 10% according to the manufacturer’s instructions. These cells were spun at 300 g for 5 minutes, and the pellets resuspended in 10ml PBS 10% before being passed through a second primed LS column and further enriched cells eluted in 5mL PBS 10% according to the manufacturer’s instructions. CD34 expression was confirmed by flow cytometry.

#### Culture of freshly isolated CD34+ cells with labeled NOV

UCB CD34+ cells were spun and the PBS 10% removed. They were then washed 3 times in 1mL StemSpan SFEM, and cultured at 37°C for 8 hours at between 1.5x10^5^ and 3x10^5^ cells/mL in SFEM supplemented with either either STF (human Stem Cell Factor 100ng/mL, human Flt3 Ligand 100ng/mL, human Thrombopoietin 100ng/mL) or STF plus AF488 labeled NOV. 10μL of labeled NOV (as eluted in protocol 1) was added *per* mL culture. All cultures were supplemented with 1x antibiotic antimycotic solution. After 8 hours the cells spun and washed three times in 1mL PBS 10% prior to staining, as described in protocol 4.

#### Identification and isolation of the NOV-Marked, NOV-Unmarked and STF-treated primitive sub-populations in UCB CD34+ cells that had been cultured in either STF or STF plus labeled NOV

The antibodies used are listed in the reagents section. Staining was performed in staining buffer (PBS 10% heat inactivated FCS, 1ng/mL Hoechst 33258).

(i) Typically, 2.5 x10^5^ – 6.0x10^5^ CD34+ cells were stained
(ii) The total volume of each staining mixture was 100μL
(iii) Lineage antibodies were all PE-Cy5 labeled, and the amounts of each in 50μL of a 4x “Lineage cocktail” are indicated in the table below.

**Table:** 

	Anti	Anti	Anti	Anti	Anti	Anti CD3	Anti	Anti	Buffer	Total
	CD14	CD16	CD56	CD19	CD2		CD123	CD235		Volume
Final titer	50	100	50	50	50	50	50	1000		
Volume in 4x Cocktail (μL)	4.0	2.0	4.0	4.0	4.0	4.0	4.0	0.2	23.8	50.0

(iv) The other antibodies used for cell staining and characterization of NOV marked sub-compartments (Figure 1) are given in the table below, which indicates the volumes of each in a single 100μL staining mixture.

**Table:** 

	NOV	Anti CD90	4x lineage cocktail	Anti CD34	Viability	Anti CD49f	Anti CD45RA	Anti CD38	Buffer	Total Volume
Fluorescence label/channel	AF488	PE	Pe-Cy5	APCCy7	Hst33258	BV650	BV711	BV785		
Final titer	0	10	4	80	0	40	60	50		
Final volume labeling mixture (μL)	0	10	25	1.3	0	2.5	1.7	2.0	57.5	100.0

(v) The other antibodies used for the isolation of NOV-Marked, NOV-unmarked and STF-treated 90+RA- cells that were subsequently used in either bulk or single-cell cultures (Figures 2A, 2B, 3B, 3C, and 4D) are given in the table below, which indicates the volumes of each in a single 100μL staining mixture.

**Table:** 

	NOV	Anti CD90	4x lineage cocktail	Anti CD34	Viability	Anti CD45RA	Anti CD38	Buffer	Total Volume
Fluorescence label/channel	AF488	PE	Pe-Cy5	APCCy7	Hst33258	BV711	BV785		
Final titer	0	10	4	80	0	60	50		
Final volume labeling mixture (μL)	0	10	25	1.3	0	1.7	2.0	60.0	100.0

(vi) Antibody staining was for 30 minutes at 4°- 8°C, and was followed by a single wash in staining buffer. Appropriate unstained, single color and FMO controls were used for compensation set-up Cell-sorting was with either a BD Aria III or a BD FACS Aria Fusion. Data analysis was with either Kaluza or FlowJo software

#### Prospective isolation of 90+RA- cells from freshly thawed UCB units

(i) UCB CD34+ cells isolated as above were stained in 100μL staining buffer.
(ii) The amount of each antibody in the staining buffer is given in the table below

**Table:** 

	Anti CD90	4x lineage cocktail	Anti CD34	Viability	Anti CD45RA	Anti CD38	Buffer	Total Volume
Fluorescence label/channel	PE	Pe-Cy5	APCCy7	Hst33258	BV711	BV785		
Final titer	10	4	80	0	60	50		
Final volume labeling mixture (μL)	10	25	1.3	0	1.7	2.0	60	100.0

The lineage cocktail was as described above.

(iii) Antibody staining was for 30 minutes at 4°- 8°C, and was followed by a single wash in staining buffer. Appropriate unstained, single color and FMO controls were used for compensation set-up Cell-sorting was with either a BD Aria III or a BD FACS Aria Fusion

#### Culture of 90+RA- cells

Freshly sorted 90+RA- cells were washed twice in 1mL StemSpan SFEM, and cultured at 37°C for 8 hours at between 5x10^3^ and 1.5x10^4^ cells/mL in SFEM supplemented with either either human Stem Cell Factor 100ng/mL, human Flt3 Ligand 100ng/mL, human Thrombopoietin 100ng/mL (STF cultures) or human Stem Cell Factor 100ng/mL, human Flt3 Ligand 100ng/mL, human Thrombopoietin 100ng/mL and human NOV 750ng/mL (NOV+STF cultures). In experiments where anti CD49f was used to block the action of NOV (Figure 2F), 2.5μg of the antibody was added to STF cultures, and the cells incubated for 1-2 minutes at room temperature before 750ng/mL NOV was added. All cultures were supplemented with 1x antibiotic antimycotic solution.

#### Single cell expansion cultures of NOV-Marked and STF-treated 90+RA- cells

Cells were sorted directly into 96 well round bottomed plates each containing 100μL Myelocult H5100 plus human Stem Cell Factor 100ng/mL, human Flt3 Ligand 100ng/mL, human Thrombopoietin 100ng/mL (STF cultures). Cultures were supplemented with 1x antibiotic antimycotic solution.

#### Long-term culture initiating cell (LTC-IC) assays

All cultures were supplemented with 1x antibiotic antimycotic solution.

(i) Humanized mouse stromal feeder cell lines M210B4 (IL-3, G-SCF) and SI/SI (IL-3, SCF) were purchased from Stem Cell Technologies, and maintained in either RPMI/10% heat inactivated FCS (M210B4) or DMEM 10% (S1/S1) as *per* the product information sheet (https://cdn.stemcell.com/media/files/pis/29301-PIS_1_0_2.pdf).
(ii) Both lines were selected for 4-5 days with G418 and Hygromycin at the recommended concentrations at every 3^rd^ passage.
(iii) All LTC-IC experiments (either bulk limiting dilution assays or single cell studies) were performed in gelatinized 96 well flat-bottomed tissue culture plates.
(iv) Gelatinization was achieved by adding 100μL 0.1% gelatin/well and leaving the plates to stand for 4 hours at room temperature. The gelatin solution was removed 30 - 60 minutes before irradiated stromal cells were added.
(v) A 1:1 mixture of M210B4 and S1/S1 was irradiated (80G) in 10mL Myelocult H5100 supplemented with 10^−6^ M Hydrocortisone. The cells were placed in 50mL centrifuge tubes for the irradiation using an X-Ray source. Irradiated stromal cells were then diluted to 50 cells/μL and 100μL added to each well of the gelatinized 96 well plates. Thus, each well contained 5000 stromal cells in total – 2500 of each type.
(vi) Freshly prepared plates were incubated for at least 12 hours at 37°C before use. Unused plates were discarded after 7 days.
(vii) In LDA studies of 90+RA- cells, doses of 80, 40, 20, 10, 5, 2 and 1 cell/well were used. Between 12 and 24 wells were inoculated at each dose. In LDA studies of CD34+ cells fractionated by CFSE fluorescence, the doses/well were 400, 200, 100 and 50. In single cell LTC-IC experiments with NOV-marked or STF-treated 90+RA- cells, the cells were sorted directly into LTC-IC plates.
(viii) After plating the LTC-IC medium was supplemented every week by removing 50μL medium and replacing with 60μL fresh 10mL Myelocult H5100 supplemented with 10^−6^ M Hydrocortisone.
(ix) After 5 weeks, the plates were spun at 100 g for 5 minutes, and all of the culture medium in each well removed, to be replaced with 75μL MethoCult H4435 “Enriched.”
(x) After 2 weeks, colony formation was assessed; the presence of at least one colony in a well indicated a positive result.
(xi) LTC-IC frequencies were computed using extreme limiting dilution analysis (ELDA) (http://bioinf.wehi.edu.au/software/elda/; Hu and Smyth, 2009).

#### CFSE staining

(i) Prospectively isolated CD34+ cells isolated from a single UCB unit (see protocol 2) were rinsed 3 times in 5mL DPBS (no serum, spun at 300 g between rinses), and then re-suspended in 1.05mL DPBS (no serum). 50μL were retained and the remainder (i.e., 1mL) used for CFSE staining.
(ii) A single tube of desiccated CFSE from the Cell Trace CFSE Proliferation Kit was reconstituted in 18μL DMSO as directed by the manufacturer, and 1μL added to 1mL of cells.
(iii) After 20 minutes’ incubation at room temperature (protected from light), the CFSE was quenched with 5mL IMDM 10%, the cells were spun at 300 g for 5 minutes, and then washed 3 times in SFEM (without cytokines).
(iv) Baseline (t = 0) CFSE staining was confirmed by comparison with the retained aliquot of cells (9(i) above).
(v) A 50μL aliquot of the CFSE-labeled cells was fixed with 5μL buffer 1 from the Perfix nc no centrifuge intracellular staining kit, and stored at 4°C until required.
(vi) The remaining labeled cells were then cultured in either NOV+STF or STF alone (cytokine concentrations as in protocol 6)
(vii) After 6 days, the cells were washed in staining buffer (protocol 4) prior to cell sorting. The aliquot of (t = 0) fixed cells (protocol 9(v) was used as a control to set the fluorescence of undivided cells

#### Measurement of cellular ROS with DCFDA

DCFDA was reconstituted immediately before use at a concentration of 20mM in DMSO. 90+RA- cells were cultured in either NOV+STF or STF for 8 hours (protocol 6). The cultures were then supplemented with 2μL 20mM DCFDA (final concentration 4μM) and returned to the incubator for 30 minutes before analysis by flow cytometry. Fluorescence was assessed directly, without washing the cells.

#### c-Myc staining

CD34+ cells isolated from a single UCB unit were cultured in NOV (750ng/mL) + STF or STF alone for 24 hours. In experiments involving CellROX green (Figure S3B), the cultures were supplemented with 5μM Cell ROX green for the final 30 minutes. The cultures were washed once in PBS 10% and then re-suspended in 100μL PBS 10%. The cells were fixed with 15μL Buffer 1 from the Perfix nc no centrifuge intracellular staining kit for 15 minutes at room temperature. 300μL of buffer 2 from the kit (permeabilizing reagent) followed by 5μL anti c-Myc DL650 and 5μL anti CD90 PE. Staining ws for 30 m minutes (protected from light). Samples were analyzed directly (without further washes).

#### Preparation of RNA and RNASeq

6,000 – 20,000 cells were harvested into 1 mL Trizol reagent (Invitrogen), snap frozen in dry ice and stored at −80°C. To prepare total RNA, the samples were thawed at RT and 200 μL of chloroform was added to each. Following a centrifugation step, RNA was isolated from the aqueous phase and precipitated through the addition of equal volumes of isopropanol supplemented with 20 μg linear polyacrylamide. Samples were washed twice in 80% ethanol (first wash over night at 4°C, second wash 5 minutes at RT). RNA pellets were re-suspended in 3-15 μL of diethylpyrocarbonate (DEPC)-treated water. RNA integrity and quantity was determined by loading of 0.5-1ul on an Agilent Bioanalyser RNA 6,000 pico chip. 2 ng total RNA was used for first strand synthesis with the ultra-low input SmartERv3 kit (Takara Clontech) followed by 13-16 cycles of PCR amplification (according to manufacturers’ instructions). cDNA was purified on Agencourt AMPureXP magnetic beads, washed twice with fresh 80% ethanol and eluted in 17 μL elution buffer. 1 μL cDNA was quantified with Qubit dsDNA HS (Molecular Probes) and checked on an Agilent Bioanalyser high sensitivity DNA chip. Sequencing libraries were produced from 150pg input cDNA using Illumina Nextera XT library preparation kit. Tagmentation time was 5mins, followed by 13-16 cycles of amplification. Libraries were then pooled and sequenced on Illumina® HiSeq using 76bp paired end kits as per manufacturer’s instructions.

#### Hexokinase II staining

90+RA- cells that had been cultured in either NOV (750ng/mL) + STF or STF alone were fixed in 1% paraformaldehyde (aqueous solution) for 60 minutes and then washed in PBS. At this point thery were stored at 4°C until required. They were pre-blocked in PBS/0.01% Triton X-100/10% Donkey serum for 30 minutes at room temperature and then incubated overnight at 4°C in PBS/0.01% Triton X-100/2% Donkey serum with anti HK II (1:20) or polyclonal rabbit IgG (negative control). The following day the cells were washed once in PBS and incubated for 45 minutes at room temperature with donkey anti-rabbit AF594. The cells were then mounted on glass culture slides in Vectorshield medium supplemented with DAPI. Images were acquired with the Perkin Elmer Ultraview Spinning Disc confocal microscope system using Volocity Acquisition and image analysis software.

#### Bioinformatics

All sequencing data was assessed to detect sequencing failures using FASTQC and lower quality reads were filtered or trimmed using *TrimGalore* (https://github.com/FelixKrueger/TrimGalore). Outlier samples containing low sequencing coverage or high duplication rates were discarded. RNaseq samples were mapped to the human reference GRCh38 using *tophat2* (https://ccb.jhu.edu/software/tophat/index.shtml). Analyses were performed within the R statistical computing framework, version 3.5 using packages from *BioConductor version 3.7* (https://Bioconductor.org). Data was combined into a per-gene count matrix using featureCounts from the subread package. The *DEseq2* BioConductor package was used for outlier detection, normalization and, differential gene expression analyses. All downstream analyses used Rlog transformed data.

#### Bone marrow reconstitution assays

(i) UCB CD34+ cells were transplanted by tail vein injection into 8-12week old male or female NSG mice that had been sub-lethally irradiated.
(ii) Sub-lethal irradiation was by a single dose of 375 cGy administered < 24 hours before transplantation.
(iii) In order to minimize adverse effects of irradiation, acid water was administered for a week prior to irradiation, and Baytril (re-suspended at 25.5 mg/kg in the drinking water) for the 2 weeks following it. Xenograft recipients were monitored weekly for weight loss, and an in-house scoring system was used to monitor for signs of ill health.
(iv) UCB CD34+ cells that were cultured prior to primary engraftment studies were washed twice in 1mL StemSpan SFEM, and then cultured at 37°C for 8 hours at between 5x10^3^ and 1.5x10^4^ cells/mL in SFEM supplemented with either either human Stem Cell Factor 100ng/mL, human Flt3 Ligand 100ng/mL, human Thrombopoietin 100ng/mL (STF cultures) or human Stem Cell Factor 100ng/mL, human Flt3 Ligand 100ng/mL, human Thrombopoietin 100ng/mL and human NOV 750ng/mL (NOV+STF cultures). After this, they were washed 3 times in PBS 0.5% FBS before counting. UCB CD34+ cells that were used in primary engraftment studies without prior culture were washed 3 times in PBS 0.5% FBS before counting. The appropriate dose of cells was administered in 150μL PBS 0.5% FBS.
(v) Primary recipients were culled after 16 weeks. Bone marrow was extracted from both femora, both tibiae, and both ilia, which were crushed in a pestle and mortar. The bone marrow cells were resuspended in 3mL IMDM 10% and collected by passing through a CellTrics 30μM sterile cell filter.
(vii) 1.5mL of the bone marrow cells were spun at 300 g for 5 minutes, re-suspended in 1x RBC Lysis Buffer and left at room temperature for 10 minutes before being re-spun and re-suspended in 2mL staining buffer.
(vii) The percentage of human engraftment was evaluated by flow cytometry (hCD45/ (hCD45^+^ + mCD45^+^)), and mice in which at least 0.1% of BM hematopoietic cells were hCD45+ were analyzed with antihuman CD34, CD33, CD235 and CD19 antibodies, and multilineage engraftment was deemed to have occurred if both CD33+ and CD19+ cells each accounted for at least 1% of all human cells.
(ix) The identification and enumeration of 90+RA- and 90-RA- cells in primary recipients was as outlined in protocol 5, except that anti human CD45 was also used.

Thus, volumes (μL) of each component of the final antibody mixture was as set out in the table below:

**Table:** 

	Anti CD90	4x lineage cocktail	Anti CD45	Anti CD34	Viability	Anti CD45RA	Anti CD38	Buffer	Total Volume
Fluorescence label/channel	PE	Pe-Cy5	AF700	APCCy7	Hst33258	BV711	BV785		
Final titer	10	4	160	80	0	60	50		
Final volume labeling mixture (μL)	10	25	0.6	1.3	0	1.7	2.0	59.4	100.0

(x) Secondary transplantation was only performed if primary engraftment was seen. In general, secondary recipients were irradiated as soon as primary engraftment data were available, and secondary transplantations performed the following morning. Primary recipient bone marrow destined for secondary transplantation was stored overnight in IMDM 10% supplemented with 1x antibiotic antimycotic solution. The cells were then washed and approximately 10 million cells from each primary recipient were transplanted by tail vein injection in 150μL PBS 0.5% FBS. Secondary engraftment was assessed after a further 16 weeks

### Quantification and Statistical Analysis

GraphPad Prism was used for all statistical analyses except for RNA-seq, which was by *DEseq2* BioConductor, and LDA (Poisson analysis), which was by ELDA. Unless otherwise indicated, mean ± SEM values are reported in the legends to graphs, in Figures 1, 2, 3, 4, and 5, n indicates the number of replicates, each replicate uses cells from a separate UCB unit. Replicate measurements were assumed to fall within a normal distribution. No data were excluded. Statistical significance was determined with Student t tests.

### Data and Code Availability

RNASeq data has been deposited at the European Genome-phenome Archive (EGA), which is hosted by the EBI and the CRG, accession number EGAS00001003979.

## Supplemental Information

Document S1. Figures S1–S6

Document S2. Article plus Supplemental Information
