# Supplementary material for: Nov/CCN3 Enhances Cord Blood Engraftment by Rapidly Recruiting Latent Human Stem Cell Activity
Source: Cell Stem Cell. 2020 Apr 2;26(4):527–541.e8. doi: 10.1016/j.stem.2020.02.012 (PMC7118368; doi:10.1016/j.stem.2020.02.012)
Supplement: Document S1. Figures S1–S6 [file mmc1.pdf]

Cell Stem Cell, Volume *26*

Supplemental Information

Nov/CCN3 Enhances Cord Blood Engraftment by Rapidly Recruiting Latent Human Stem Cell Activity

Rajeev Gupta, Virginia Turati, Duncan Brian, Craig Thrussel, Barry Wilbourn, Gillian May, and Tariq Enver

Figure S1 Related to Figure 1

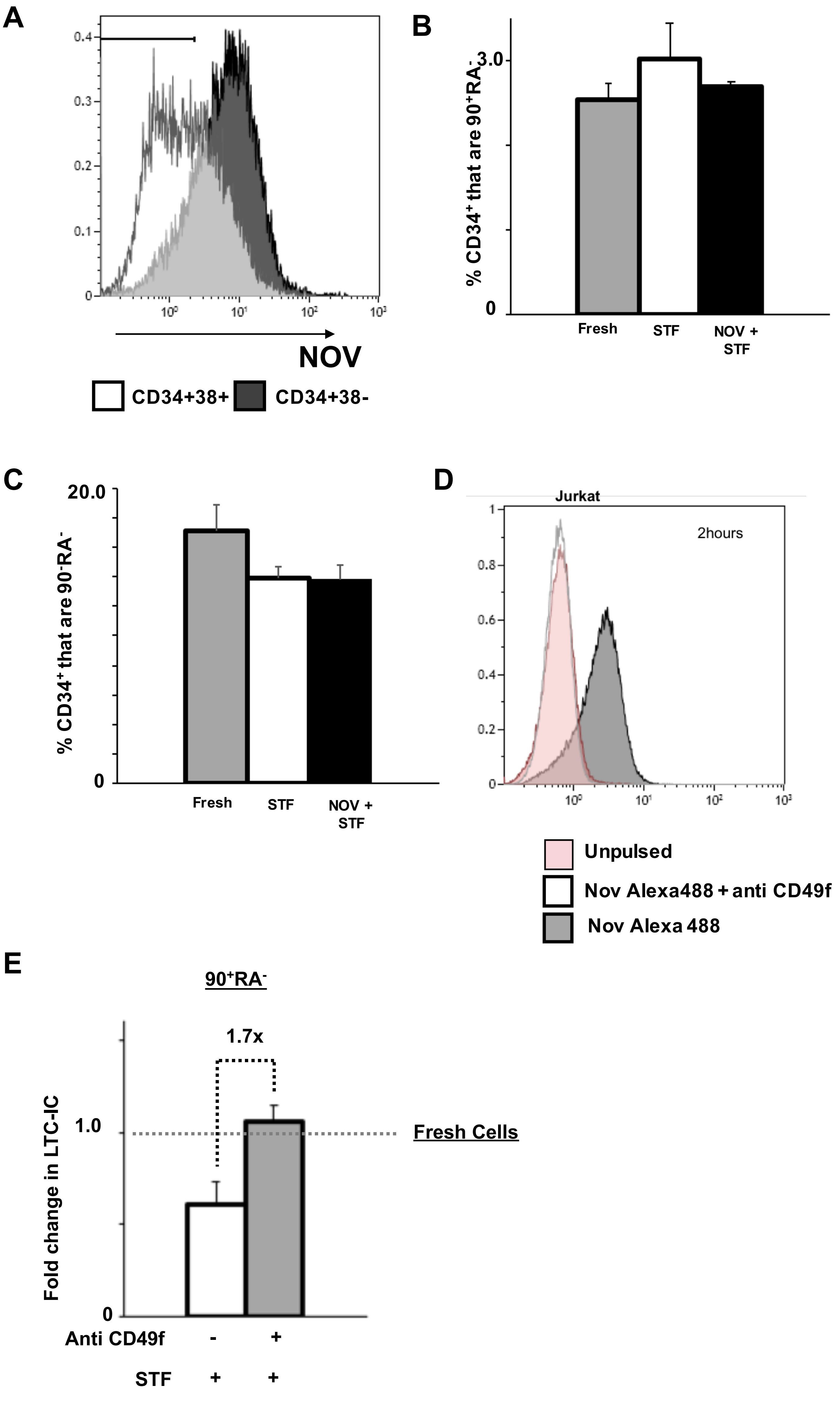

Figure S1 Related to Figure 1

(A) Flow cytometry overlay histogram showing binding of labelled NOV to UCB CD34+38+ and CD34+38- cells. Solid line, staining control. (B) Fraction of UCB CD34+ cells that are CD38-Lin-45RA- 90+ or (C) CD38-Lin-45RA- 90- before (fresh) and after incubation with either STF or NOV+STF. Comparison of three separate UCB donations, p= NS (t-test). (D) Overlay histogram showing the extent of labelling of Jurkat cells after incubation with Alexa-488-labelled NOV protein, and blocking after pre-incubation with anti CD49f antibody for 2 hours. (E) Fold changes in absolute numbers of LTC-IC in STF and anti-CD49f+ STF treated 90+RA- cells. The number of LTC-IC in the unmanipulated cells inoculated into either culture is normalized to 1.0 (dashed line) and the mean fold-change + SEM in the absolute number of LTC-IC at the end of each culture calculated relative to this. n=5 UCB donations, mean + SEM, p (STF *vs* anti CD49f+STF) = 0.07.

Figure S2 related to Figure 3

A

Numbers of LTC-IC in each single cell experiment

90+RA- single cells	STF	NOV marked
CB 1	11/192	18/192
CB 2	9/192	31/288
CB 3	9/288	17/288
CB 4	7/288	17/288
Mean	3.79/100	7.98/100
SD	2.44	1.59
SEM	0.79	1.22

90+RA- single cells	STF	NOV marked
CB 5	42/288	61/288

B

Numbers of LTC-IC in each single cell experiment

90+RA- single cells	STF	NOV marked
CB 1	5/192	11/192
CB 2	6/288	10/288
CB 3	1/288	4/288
CB 4	1/288	4/288
Mean	1.33/100	3.00/100
SD	1.20	2.05
SEM	0.60	1.03

90+RA- single cells	STF	NOV marked
CB 5	10/288	21/288

Figure S2 Related to Figure 3 and Figure 4

(A) CD34+ cells from 4UCB donations (CB1-CB4) were incubated in either STF or labelled NOV plus STF for 8 hours, and NOV-marked and STF treated 90+RA- were deposited as single cells onto LTC-IC stroma in 96 well format. The number of wells scoring as LTC-IC and the mean number of LTC-IC/100 cells are provided. A fifth UCB unit (CB5) was also studied, but the methylcellulose used in this experiment was from a different manufacturer. Nonetheless the trend of the results is the same. (B) CD34+ cells from 4 individual UCB donations (CB1-CB4) were incubated in either STF or STF plus labelled NOV for 8 hours and NOV-marked and STF treated 90+RA- were deposited as single cells into stem cell expansion medium supplemented with STF in 96 well format. After 14 days, the entire progeny of each cell was inoculated into LTC-IC. The number of wells scoring as LTC-IC and the mean number of LTC-IC/100 cells are provided. A fifth UCB donation (CB5) was also studied, but the methylcellulose used in this experiment was from a different manufacturer. Nonetheless the trend of the results is the same.

Figure S3 related to Figure 4 and Figure 5

A

% cells that are CD34+ after 6 days

	NOV + STF	STF
Div 0	87%	88%
Div1	88%	88%
Div2	86%	87%
Div3	80%	81%
Div4	63%	69%

B

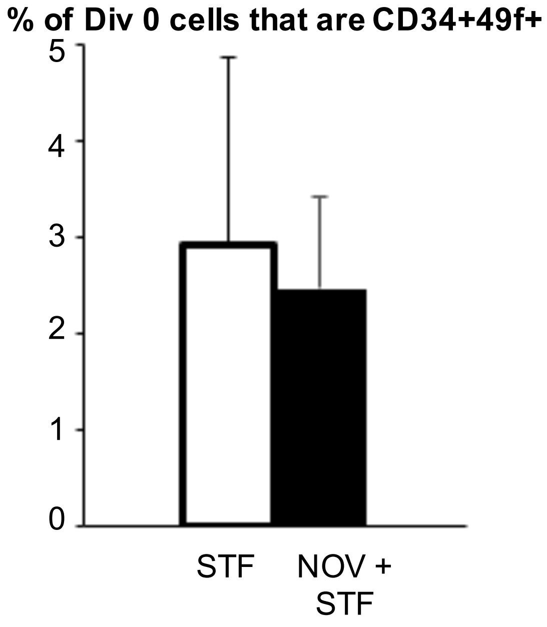

C

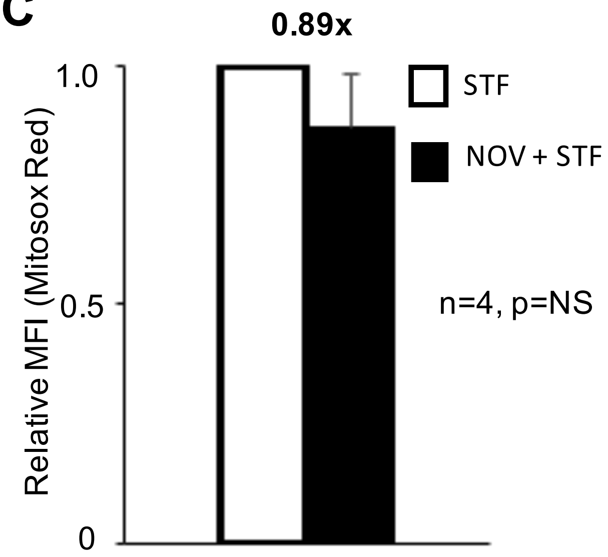

D

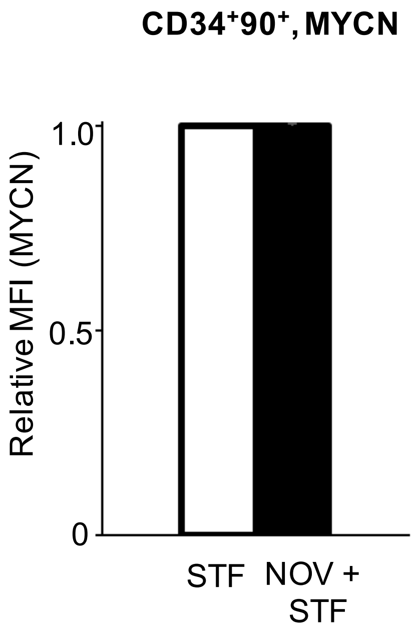

E

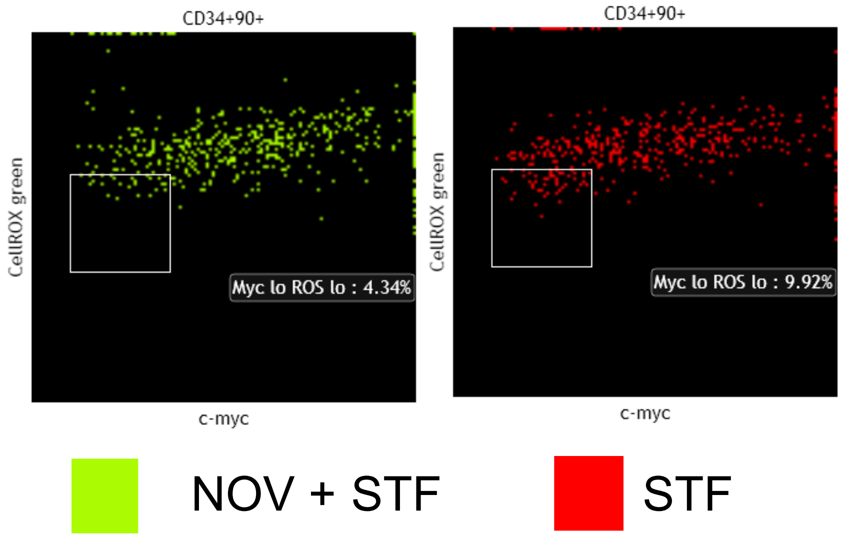

Figure S3 Related to Figure 4 and Figure 5

UCB derived CD34+ cells were labelled with CFSE and then cultured for 6 days in either STF or NOV+STF. (A) The percentages of cells that are CD34+ in those compartments that had undergone 0, 1, 2, 3 or 4 divisions in each culture condition. (B) The percentages of CD34+49f+ cells in the undivided (0 divisions) compartment, +SEM, n=2, p=NS. (C) MFI of Mitosox Red staining in CD34+90+ cells cultured in either STF or NOV+STF. n=4, mean + SEM, p (STF *vs* NOV+STF) = 0.32 (D) MFI of MYCN staining in UCB derived CD34+90+ cells cultured in either STF or STF + NOV. n=2, mean + SEM, p (STF *vs* NOV plus STF) = NS. (EB) Representative flow cytometry density plot of CD34+90+ cells cultured in either STF or STF plus NOV showing followed by staining with both CellROX Green (a fixable marker for ROS) and anti c-MYC antibody.

Figure S4 related to Figure 5

A

Gene set	ES	NES	NOM p-val	FDR q-val	FWER p-val
ZHANG_PROLIFERATING_VS_QUIESCENT	-0.28	-2.33	0.002	0.008	0.708
GRAHAM_CML_QUIESCENT_VS_NORMAL_QUIESCENT_DN	-0.11	-0.91	0.560	0.734	1.000
GRAHAM_NORMAL_QUIESCENT_VS_NORMAL_DIVIDING_DN	-0.19	-2.10	0.002	0.026	0.992
GRAHAM_CML_DIVIDING_VS_NORMAL_QUIESCENT_UP	-0.12	-1.87	0.004	0.071	1.000

B

Gene set	ES	NES	NOM p-val	FDR q-val	FWER p-val
REACTOME_GLYCOLYSIS	-0.25	-1.52	0.060	0.229	1.000
HALLMARK_GLYCOLYSIS	-0.08	-1.38	0.115	0.301	0.949

C

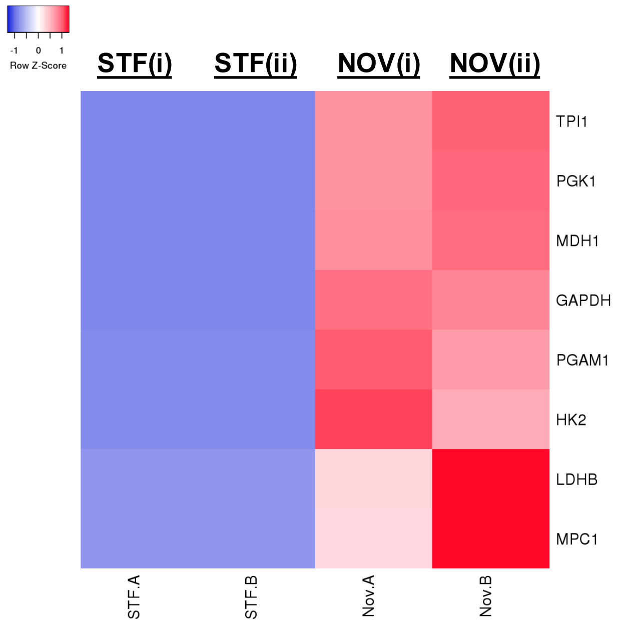

D

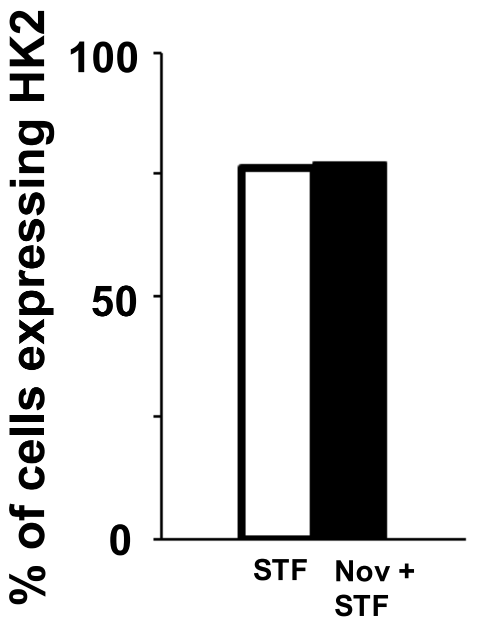

E

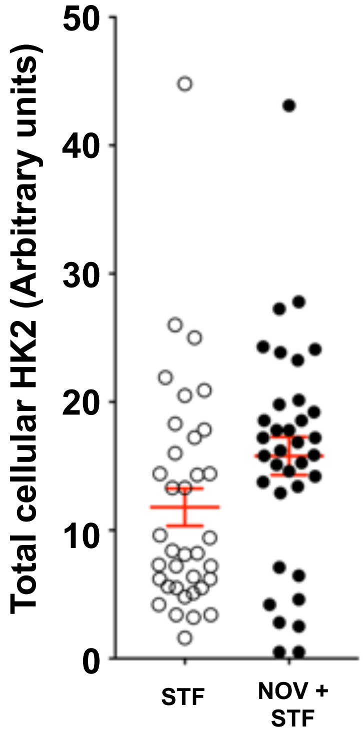

F

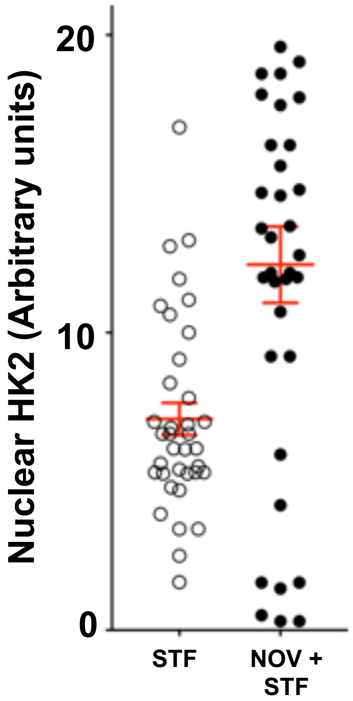

Figure S4 Related to Figure 5

Tables showing results of GSEA for transcripts associated with cellular quiescence (A) and glycolysis (B) between the RNASeq profiles of 90+RA- cells cultured in either STF or NOV+STF for 8 hours. There is no enrichment in the expression of markers of either cellular process. (ES enrichment score, NES normalized enrichment score, FDR false discovery rate, FWER family wise error rate). (C) Heat map showing expression of genes encoding glycolytic enzymes whose expression was upregulated in 90+RA- cells in both experiments. NOV+STF treated cells are labelled as "NOV". (D) Percentages of 90+RA- cells that have been cultured in either STF or NOV+STF in which Hexokinase 2 protein (HK2) can be detected by fluorescence microscopy. HK2 was detected in 38/49 STF and 37/47 STF plus NOV treated cells (data from 2 UCB units). (E, F) The relative levels of total and nuclear HK2 recorded as arbitrary units of fluorescence in 90+RA- cells. Red bars = mean +/- SEM. Mean total HK2 STF 11.8, STF plus NOV 15.8 p=0.06. Mean nuclear HK2 STF 7.1, STF plus NOV 12.2 p=0.0004.

Figure S5 related to Figure 6

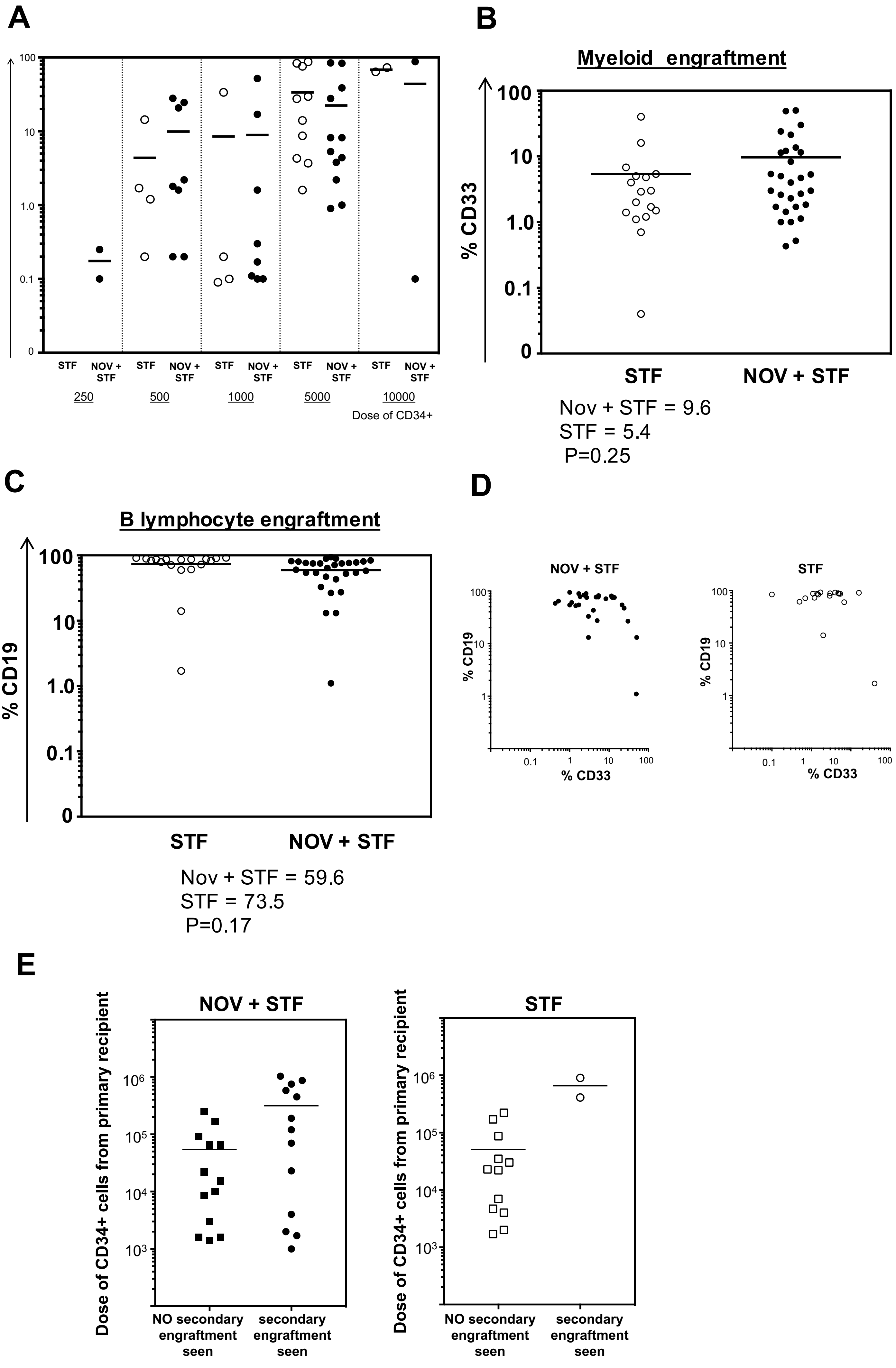

Figure S5 Related to Figure 6

(A) Total human engraftment in 1°NSG recipients 16 weeks after transplantation with the indicated doses of UCB CD34+ cells cultured for 8h in either STF or NOV+STF. Percentage myeloid (CD33+) (B) and B lymphoid (CD19+) (C) reconstitution in all primary recipients showing human engraftment, black horizontal bar shows the mean (D) Relative levels of myeloid versus lymphoid human reconstitution in individual recipients of NOV+STF (left) and STF (right) treated CD34+ cells. Each circle represents a single recipient. (E) Doses of human CD34+ cells in the bone marrow of NOV+STF (left) and STF (right) treated primary recipients given to each secondary recipient. The doses of cells given to mice that did and did not show secondary engraftment are indicated.

Figure S6 related to Figure 6 and Figure 7

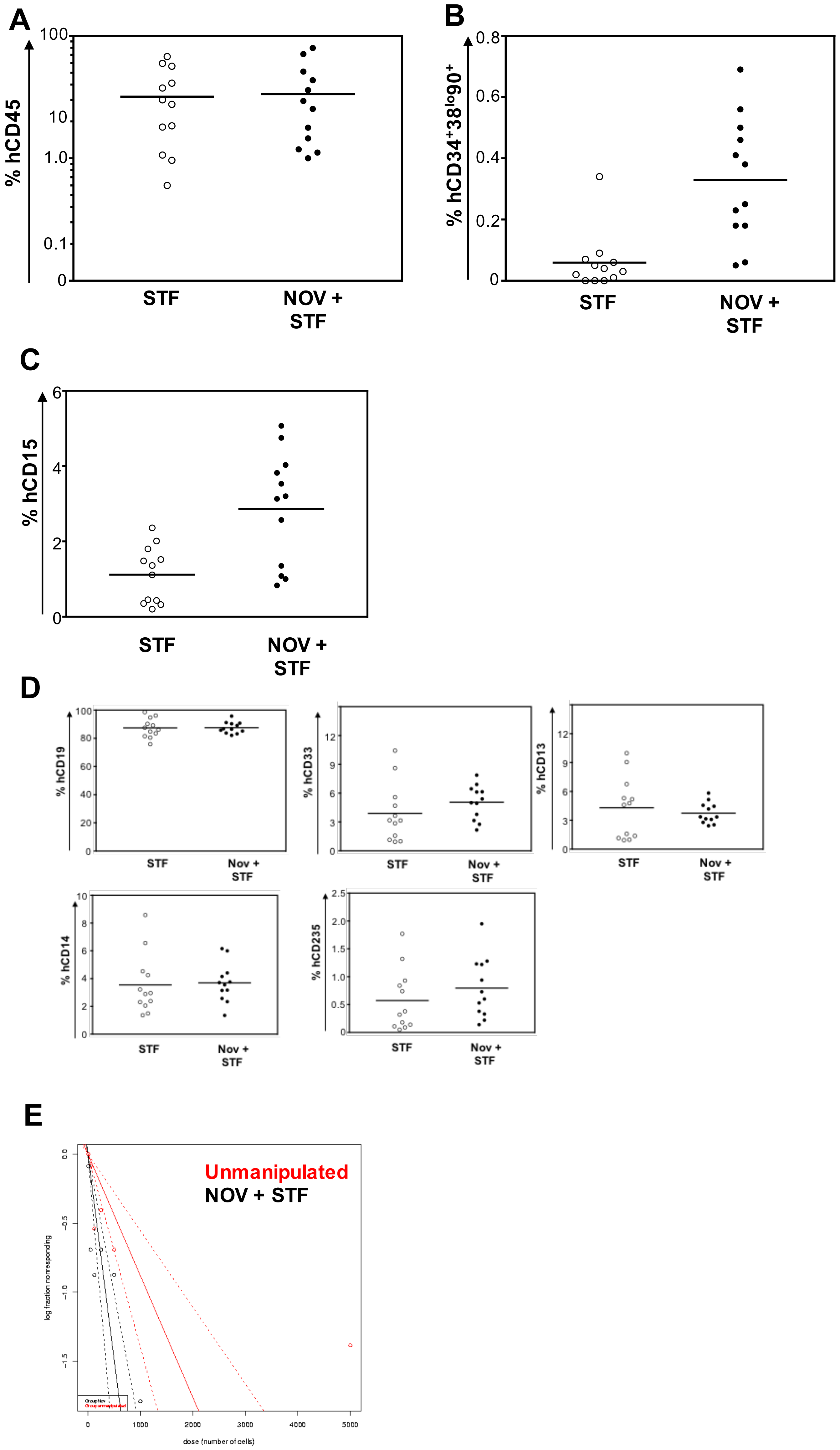

Figure S6 Related to Figure 7

Human engraftment in BM of 1°NSG recipients of 10000 UCB CD34+ cells after 8 weeks. (A) Total human engraftment (Mean STF 31.9%, NOV+STF 33.4%p=NS) (B) Percentage of human cells that are $CD34^{+}38^{lo}90^{+}$ (Mean STF 0.06%, NOV 0.33%, p=0.0002) (C) Percentage of human cells that are $CD15^{+}$ (Mean STF 1.2%, NOV+STF 2.9%, p=0.0022). (D) Percentage of human cells that are $CD19^{+}$ (Mean STF 87.4, NOV+STF 87.5%, % p=NS), CD33+ (Mean STF 3.9%, NOV+STF 5.1%,p=NS), $CD13^{+}$ (Mean STF 4.3%, NOV+STF 3.7%, p=NS), $CD14^{+}$ (Mean STF 3.6%, NOV+STF 3.7%, p=NS) and $CD235^{+}$ (Mean STF 0.6% NOV+STF 0.8%, p=NS). (E) graph of LTC-IC frequencies in a single unit of mobilized peripheral blood CD34+ cells from a patient with a non-haematological disorder before (unmanipulated, red) and after incubation with NOV+STF (black). NOV+STF 1/336, unmanipulated 1/1134, p (NOV+STF *v* unmanipulated) = <0.01.
